# A molecular complex of Ca_v_1.2/CaMKK2/CaMK1a in caveolae is responsible for vascular remodeling via excitation–transcription coupling

**DOI:** 10.1073/pnas.2117435119

**Published:** 2022-04-11

**Authors:** Yoshiaki Suzuki, Takumi Ozawa, Tomo Kurata, Nanami Nakajima, Gerald W. Zamponi, Wayne R. Giles, Yuji Imaizumi, Hisao Yamamura

**Affiliations:** ^a^Department of Molecular and Cellular Pharmacology, Graduate School of Pharmaceutical Sciences, Nagoya City University, Nagoya 467-8603 Japan;; ^b^Department of Physiology & Pharmacology, Cumming School of Medicine, University of Calgary, Calgary, AB T2N 4N1, Canada;; ^c^Hotchkiss Brain Institute, Alberta Children‘s Hospital Research Institute, Cumming School of Medicine, University of Calgary, Calgary, AB T2N 4N1, Canada

**Keywords:** excitation–transcription coupling, vascular smooth muscle cell, voltage-dependent Ca^2+^ channel, caveolin-1, vascular remodeling

## Abstract

Elevation of intracellular Ca^2+^ concentration ([Ca^2+^]_i_) activates Ca^2+^/calmodulin-dependent kinases (CaMK) and promotes gene transcription. This signaling pathway is referred to as excitation–transcription (E-T) coupling. Although vascular myocytes can exhibit E-T coupling, the molecular mechanisms and physiological/pathological roles are unknown. Multiscale analysis spanning from single molecules to whole organisms has revealed essential steps in mouse vascular myocyte E-T coupling. Upon a depolarizing stimulus, Ca^2+^ influx through Ca_v_1.2 voltage-dependent Ca^2+^ channels activates CaMKK2 and CaMK1a, resulting in intranuclear CREB phosphorylation. Within caveolae, the formation of a molecular complex of Ca_v_1.2/CaMKK2/CaMK1a is promoted in vascular myocytes. Live imaging using a genetically encoded Ca^2+^ indicator revealed direct activation of CaMKK2 by Ca^2+^ influx through Ca_v_1.2 localized to caveolae. CaMK1a is phosphorylated by CaMKK2 at caveolae and translocated to the nucleus upon membrane depolarization. In addition, sustained depolarization of a mesenteric artery preparation induced genes related to chemotaxis, leukocyte adhesion, and inflammation, and these changes were reversed by inhibitors of Ca_v_1.2, CaMKK2, and CaMK, or disruption of caveolae. In the context of pathophysiology, when the mesenteric artery was loaded by high pressure in vivo, we observed CREB phosphorylation in myocytes, macrophage accumulation at adventitia, and an increase in thickness and cross-sectional area of the tunica media. These changes were reduced in caveolin1-knockout mice or in mice treated with the CaMKK2 inhibitor STO609. In summary, E-T coupling depends on Ca_v_1.2/CaMKK2/CaMK1a localized to caveolae, and this complex converts [Ca^2+^]_i_ changes into gene transcription. This ultimately leads to macrophage accumulation and media remodeling for adaptation to increased circumferential stretch.

## Significance

Excitation–transcription (E-T) coupling can initiate and modulate essential physiological or pathological responses in cells, such as neurons and cardiac myocytes. Although vascular myocytes also exhibit E-T coupling in response to membrane depolarization, the underlying molecular mechanisms are unknown. Our study reveals that E-T coupling in vascular myocytes converts intracellular Ca^2+^ signals into selective gene transcription related to chemotaxis, leukocyte adhesion, and inflammation. Our discovery identifies a mechanism for vascular remodeling as an adaptation to increased circumferential stretch.

Changes in intracellular Ca^2+^ concentration ([Ca^2+^]_i_) are involved in a large number of biological responses, such as neurotransmission, muscle contraction, and hormone secretion (1, 2). In cardiac myocytes, opening of voltage-dependent Ca^2+^ channels (VDCCs) in response to membrane depolarization causes Ca^2+^-induced Ca^2+^ release (CICR) mediated by nearby ryanodine receptors (RyRs) (3). CICR increases global [Ca^2+^]_i_ and this initiates muscle contraction: that is, excitation–contraction (E-C) coupling. In contrast, local Ca^2+^ release from the RyRs (Ca^2+^ sparks) in vascular smooth muscle cells activates nearby large conductance Ca^2+^-activated K^+^ (BK) channels in the absence of global [Ca^2+^]_i_ increases, and this generates spontaneous transient outward currents. Spontaneous transient outward currents lead to membrane hyperpolarization, as well as a decrease in VDCC activity and vascular tone (4). This essential transduction between electrical and [Ca^2+^]_i_ signals has been shown to occur in spatially localized intracellular domains by scaffold proteins, such as caveolin (5).

An increase in [Ca^2+^]_i_ turns on Ca^2+^-dependent enzymes, resulting in activation of transcription factors and subsequent changes in gene-transcription patterns (6, 7). This scheme is often referred to as excitation–transcription (E-T) coupling. E-T coupling is one of the molecular mechanisms of synaptic plasticity or late-phase long-term potentiation in neurons (6) and is also important for myocardial remodeling (8). In smooth muscle cells, E-T coupling mechanisms directly associated with Ca^2+^ influx through VDCCs have been reported to play important roles in physiological or pathophysiological functions. The calcineurin/NFATc3 pathway can modulate the expression of several genes, including those for specific K^+^ channel subunits, such as Kv2.1, as well as the β1 subunit of BK channels, which results in angiotensin II- (9) or high glucose- (10) induced hypertension. The Rho/ROCK pathway has been reported to induce myocardin/SRF-dependent transcription of smooth muscle marker genes, such as *Myh11* and *Acta2*, and contributes to smooth muscle differentiation (11, 12). On the other hand, the Ca^2+^/calmodulin-dependent kinase (CaMK)/cyclic AMP-responsive element binding protein (CREB) pathway is activated by sustained depolarization in vascular smooth muscle (13, 14), but the detailed molecular mechanisms and physiological or pathophysiological roles are unclear.

Vascular remodeling is initiated and maintained as a compensation mechanism for various stress conditions in blood vessels. For example, it is known that the vessel diameter and the thickness of the vessel wall are changed by alteration of blood flow or transmural pressure following surgical arterial ligation (15, 16). Importantly numerous studies have revealed that macrophage accumulation and resulting inflammation play an important role in progress of vascular remodeling (16–18). However, it is unclear how macrophages are attracted to the blood vessels exposed to stress consisting of maintained pressure changes.

Previously we reported that caveolin-1 (cav1), an essential component of caveolae in smooth muscle, can support effective conversion between electrical and [Ca^2+^]_i_ signals (5, 19). We showed that this occurs due to spatially localized molecular complexes of VDCCs, RyRs, and BK channels in caveolae, thus forming a Ca^2+^ microdomain that can tune vascular tone (5, 19). In the present study, we significantly extend these findings by revealing that the cav1 forms a molecular complex consisting of Ca_v_1.2/CaMKK2/CaMK1a and induces CREB phosphorylation in vascular myocytes. We also reveal that a sustained depolarization stimulus induces gene transcription related to chemotaxis and inflammation in mesenteric artery. Finally, our data from a mesenteric artery ligation model shows that cav1 and CaMKK2 are important for: 1) CREB phosphorylation in vascular myocytes, 2) macrophage accumulation in adventitia, and 3) vascular remodeling. In summary, E-T coupling in vascular myocytes can modulate macrophage migration and subsequent inflammation, thus altering the vascular structure.

## Results

### Ca_v_1.2/CaMKK2/CaMK1a Pathway Is Responsible for E-T Coupling in Vascular Myocytes.

**Fig. 1.**
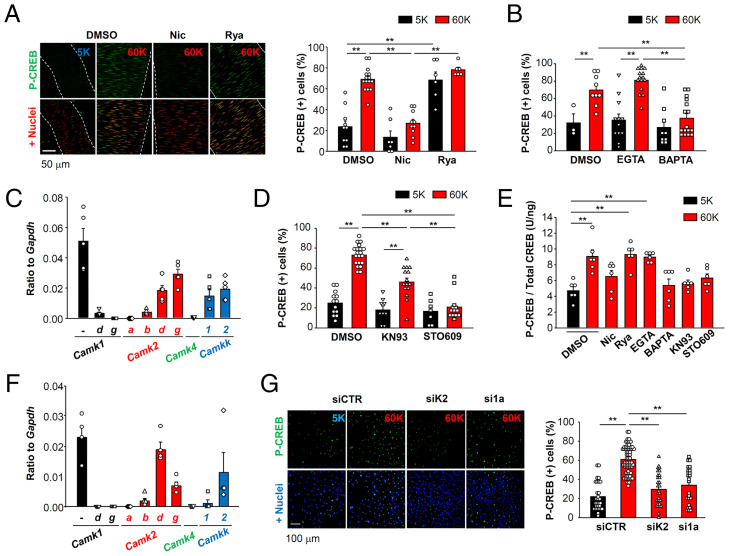
Ca^2+^ influx through Ca_v_1.2 channels can cause CREB phosphorylation by activating CaMKK2 and CaMK1a in vascular myocytes. (*A*) Mesenteric artery myocytes were depolarized for 30 min by applying a 60 mM K^+^ solution (60K) containing DMSO, 1 μM Nic, or 10 μM Rya. The arteries stained with P-CREB antibody (green) and TO-PRO3 (red) were analyzed using a confocal microscope (*Left*). In the present study, second- and third-order mesenteric arteries were utilized unless otherwise stated. Smooth muscle nuclei colocalized with P-CREB signals were detected and quantified, and the ratio of P-CREB (+) nuclei to total nuclei was calculated (*Right*). Data were obtained from 6 to 15 arteries per group. (*B*) Mesenteric artery was pretreated with EGTA-AM or BAPTA-AM (30 μM) for 45 min, and stimulated using 60 mM K^+^ solution. Data were obtained from 3 to 16 arteries per group. (*C*) mRNA levels of CaMK family genes in mesenteric artery. Data were obtained from five mice. (*D*) Mesenteric arteries were stimulated with 60 mM K^+^ solution either with or without CaMK inhibitors (30 μM KN93 or 10 μM STO609). Data were obtained from 7 to 20 arteries per group. (*E*) The amount of P-CREB in mesenteric arteries was quantified using ELISA. The concentration of both P-CREB and total CREB in each arterial lysate were calculated and P-CREB/total CREB ratio was compared. Data were obtained from six mice per group. (*F*) mRNA levels of CaMK family genes in primary vascular myocytes are shown. Data were obtained from four independent unpassaged cultures. (*G*) Primary vascular myocytes were treated with siRNA, which targeted CaMKK2 (siK2) or CaMK1a (si1a). The vascular myocytes were preincubated with 5 mM K^+^ HEPES-buffered solution for 30 min, stimulated with 60 mM K^+^ solution for 30 min and stained with P-CREB antibody (green) and Hoechst (blue) (*Left*). The P-CREB (+) nuclei ratio per section (635 μm × 635 μm) is compared (*Right*). Data were obtained from 24 to 67 sections per group. Statistical analysis was performed using one-way (*E* and *G*) or two-way (*A*, *B*, and *D*) ANOVA followed by Dunnett or Tukey tests (**P < 0.01).

Mouse mesenteric arterial bed preparations (20) were depolarized by perfusion with 60 mM K^+^ solution for 30 min (Fig. 1*A*). This sustained depolarization caused CREB phosphorylation in nuclei in these vascular myocytes, which could be blocked by the L-type VDCC inhibitor, nicardipine (Nic, 1 μM). The RyR inhibitor ryanodine (Rya, 10 μM) did not block CREB phosphorylation in the vascular myocytes. Instead, Rya caused CREB phosphorylation in the absence of K^+^-induced depolarization due to an inhibition of BK channels that induces membrane depolarization (13). These results show that depolarizing stimuli can cause Ca^2+^ influx, probably through the dominant VDCC isoform in vascular myocytes, Ca_v_1.2, as well CREB phosphorylation in these myocytes.

Our next experiments examined whether local Ca^2+^ signaling, within Ca^2+^ microdomains, is sufficient to result in intranuclear CREB phosphorylation in vascular myocytes. Two types of Ca^2+^ chelators, EGTA and BAPTA (5, 21), were used. The fast Ca^2+^ chelator BAPTA can effectively prevent Ca^2+^ signal transmission from VDCC to its effectors within the same Ca^2+^ microdomains, while the much slower Ca^2+^ chelator EGTA cannot (7, 21). Preincubation of the mesenteric arterial bed preparations with 30 μM BAPTA-AM strongly inhibited CREB phosphorylation, but 30 μM EGTA-AM failed to show this effect (Fig. 1*B*). Thus, changes in [Ca^2+^]_i_ due to VDCC-mediated Ca^2+^ influx directly can activate Ca^2+^-sensitive molecules within presumed Ca^2+^ microdomains and this results in intranuclear CREB phosphorylation in these vascular myocytes.

Molecular mechanisms that connect local [Ca^2+^]_i_ signals and intranuclear CREB phosphorylation significantly differ depending on the target tissues being studied (8, 22). It is known that the CaMK family is responsible for CREB phosphorylation (23). qPCR analysis showed mRNA expression of CaMK1a (*Camk1*), CaMK2δ (*Camk2d*), CaMK2γ (*Camk2g*), CaMKK1 (*Camkk1*), and CaMKK2 (*Camkk2*) in mouse mesenteric artery samples (Fig. 1*C*). Within this CaMK family, CaMK2γ_A_ and CaMK2δ_B_ isoforms contain a classic nuclear localizing signal (NLS) (24). In excitatory neurons, CaMK2γ_A_ and CaMK4 are responsible for CREB phosphorylation (22). On the other hand, CaMK1γ is known to be able to shuttle CaM to the nucleus and to activate CaMK4 in parvalbumin-positive interneurons (25). In smooth muscle tissues, however, CaMK2γ_B_ and CaMK2γ_C_ but not CaMK2γ_A_ are dominant among the identified CaMK2γ splice variants (*SI Appendix*, Fig. S1*A*), as previously reported (26). CaMK1γ (*Camk1g*) mRNA level was also negligible in mesenteric arteries. In addition, neither CaMK4 (Fig. 1*C*) nor CaMK2δ_B_ mRNA (*SI Appendix*, Fig. S1*B*) could be detected in either mesenteric artery or primary vascular myocytes. Thus, vascular myocytes must utilize different intracellular signaling pathways to transmit Ca^2+^ signals to nuclei.

The nonspecific CaMK inhibitor, KN93 (30 μM), inhibited CREB phosphorylation in vascular myocytes (Fig. 1*D*). CaMKK is an upstream kinase in CaMK1 and CaMK4 signaling pathways (23, 27). Importantly, the CaMKK inhibitor, STO609 (10 μM) (28), completely inhibited CREB phosphorylation in these vascular myocytes (Fig. 1*D*). [Ca^2+^]_i_ imaging showed that neither KN93 nor STO609 had any effects on VDCC-mediated [Ca^2+^]_i_ elevations (*SI Appendix*, Fig. S2). For a more quantitative analysis of effects of the drugs on CREB phosphorylation, an ELISA was utilized using arterial lysates (Fig. 1*E*). Similar to the results from immunostaining experiments, increased CREB phosphorylation was detected in arteries stimulated with 60 mM K^+^ + DMSO, Rya, or EGTA. Taken together, these results strongly suggest that a CaMKK-CaMK pathway is essential for CREB phosphorylation in vascular myocytes.

Accordingly, we sought to identify the isoforms of CaMKK and CaMK involved in E-T coupling in these myocytes. Given the difficulty of knockdown or overexpression of exogenous proteins in native tissue, we utilized primary, unpassaged vascular myocytes from mouse aorta (29). Immunostaining showed that these primary vascular myocytes were positive for α-smooth muscle actin (*SI Appendix*, Fig. S3*A*). As expectedly, these myocytes responded to a depolarizing stimulus (60 mM K^+^ solution) with a robust increase in [Ca^2+^]_i_ (*SI Appendix*, Fig. S3 *B* and *C*). These findings strongly suggest that these primary vascular myocytes have Ca^2+^ signaling features that are common to freshly isolated myocytes. mRNA expression of CaMKK1 in these primary myocytes was not detected (Fig. 1*F*), and therefore CaMKK2 in these myocytes was silenced using specific small-interfering RNA (siRNA) (*SI Appendix*, Fig. S3*D*). As shown in Fig. 1*G*, CaMKK2 knockdown significantly decreased depolarization induced CREB phosphorylation in these primary vascular myocytes.

CaMKK2 can phosphorylate CaMK1 and CaMK4, and both of these enzymes can induce P-CREB–dependent gene expression (23, 27). Expression analyses of CaMK1 and CaMK4 revealed that abundant mRNA expression of CaMK1a could be detected in mesenteric artery samples and in the primary vascular myocytes (Fig. 1 *C* and *F*). Knockdown of CaMK1a (*SI Appendix*, Fig. S3*D*) decreased CREB phosphorylation in the primary vascular myocytes (Fig. 1*G*). Taken together, these results demonstrate that an increase in [Ca^2+^]_i_ through VDCCs activates a CaMKK2-CaMK1a-CREB pathway in vascular myocytes.

### Caveolae Are Essential for Physical and Functional Coupling between Ca_v_1.2 Channels and CaMKK2 in Vascular Myocytes.

**Fig. 2.**
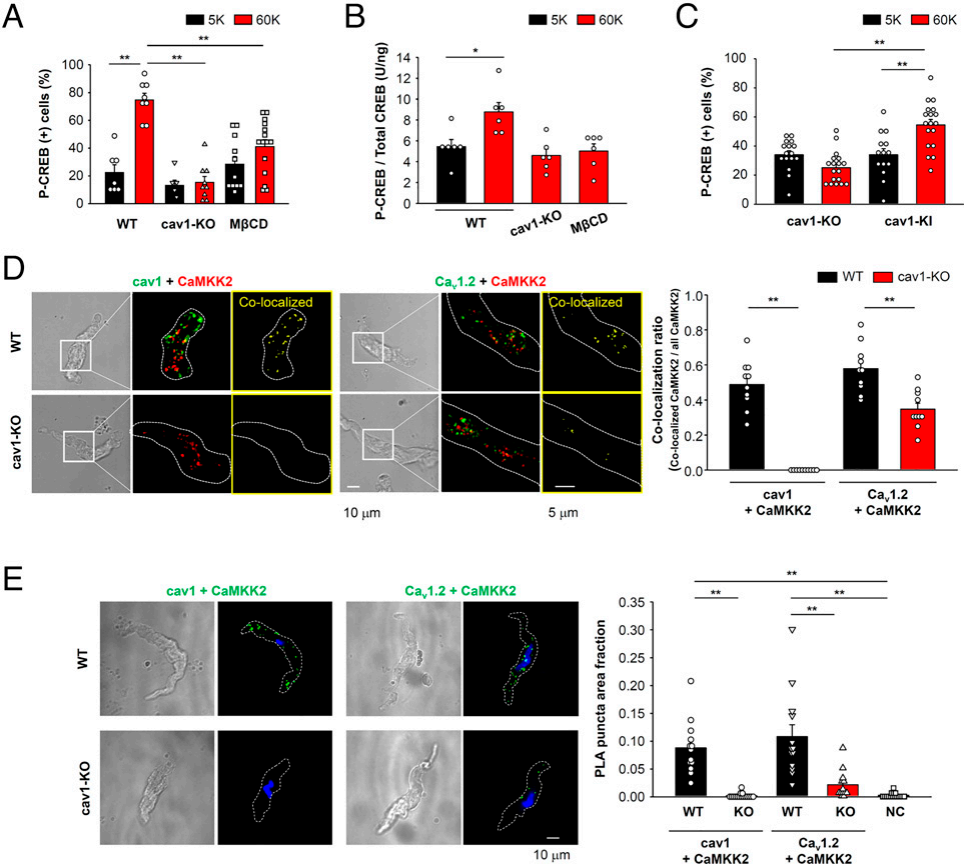
Caveolae promote CREB phosphorylation by enhancing a Ca_v_1.2-CaMKK2 complex. (*A*) Mesenteric arteries from WT or cav1-KO were stimulated with 60 mM K^+^ solution for 30 min. MβCD (10 mM) was utilized to disrupt caveolae. Data were obtained from 6 to 15 arteries per group. (*B*) The amount of P-CREB in mesenteric arteries was quantified using ELISA. The concentration of both P-CREB and total CREB in each arterial lysate were calculated and P-CREB/total CREB ratio was compared. Data were obtained from six mice per group. (*C*) Primary vascular myocytes from cav1-KO mice transfected with mock (cav1-KO) or cav1 (cav1-KI) were stimulated with 60 mM K^+^ solution after preincubation with 5 mM K^+^ solution for 30 min and the P-CREB (+) nuclei ratio was compared (14 to 18 sections per group). (*D*) Localization of cav1 (green) and CaMKK2 (red), or Ca_v_1.2 (green) and CaMKK2 (red) on the plasma membrane in freshly isolated mesenteric artery myocytes was visualized using a TIRF microscope. TIRF images obtained from the cell regions surrounded by white square in brightfield images are presented. Dashed lines indicate TIRF footprints. Fluorescence puncta from the secondary antibodies (shown as green and red in the images) were converted to binary images and colocalized puncta (shown as yellow) were extracted. The ratio of the number of colocalized puncta to the total number of CaMKK2 puncta (colocalization ratio) is compared. Data were obtained from 10 cells per group. (*E*) Proximity between cav1-CaMKK2 and Ca_v_1.2-CaMKK2 in mesenteric artery myocytes was determined using a PLA (green). Nuclei were stained with Hoechst (blue, *Left*). PLA puncta were extracted by binary image processing and the PLA puncta area was normalized to the whole cell area indicated by dashed lines (PLA puncta area fraction, *Right*). Negative control (NC) data were obtained by treating cells only with anti-CaMKK2 antibody. Data were obtained from 13 to 16 cells per group. Statistical analysis was performed using one-way (*B* and *E*) or two-way (*A* and *C*) ANOVA followed by Tukey or Dunnett tests and two-tailed *t* test (*D*) (*P < 0.05, **P < 0.01).

Since CREB phosphorylation was resistant to EGTA treatment (Fig. 1*B*), we hypothesized that Ca_v_1.2, CaMKK2 and CaMK1a form molecular complexes in or very near the plasma membrane. Cav1, a well-characterized scaffolding protein, can promote molecular coupling between Ca_v_1.2 and downstream molecules (5, 19). Therefore, the possibility that caveola/cav1 can promote Ca_v_1.2-CaMKK2 complexes was examined. In mesenteric arteries from cav1-knockout (KO) mice, a 60 mM K^+^ solution-induced depolarization failed to result in CREB phosphorylation (Fig. 2*A*), even though functional Ca_v_1.2 expression in cav1-KO myocytes was very similar to that of WT cells (5). Moreover, the mRNA levels of most CaMK isoforms were very similar between WT and cav1-KO tissues. The exception was the slight increase in CaMK2γ (*Camk2g*) mRNA (*SI Appendix*, Fig. S3*E*). Caveola disruption using methyl-β-cyclodextrin (MβCD, 10 mM) also inhibited CREB phosphorylation in mouse mesenteric artery (Fig. 2*A*). Similar results were obtained by ELISA using whole arterial lysates (Fig. 2*B*). CREB phosphorylation was not detected in primary vascular myocytes from cav1-KO, while an increase in [Ca^2+^]_i_ produced by 60 mM K^+^ stimulus was very similar between WT and cav1-KO (*SI Appendix*, Fig. S3 *B* and *C*). In contrast, cav1 overexpression in cav1-KO myocytes (*SI Appendix*, Fig. S3*F*) restored CREB phosphorylation (Fig. 2*C*). This pattern of results strongly suggests that cav1 is critical for depolarization-induced CREB phosphorylation in these vascular myocytes.

To further evaluate the functional effects of a Ca^2+^ signaling microdomain that can regulate E-T coupling, we examined intracellular localization of Ca_v_1.2, CaMKK2, and cav1 using a total internal reflection fluorescence (TIRF) microscope that provides the possibility of selective detection of fluorescence signals just beneath the plasma membrane (5, 19). Significant colocalization between cav1 and CaMKK2 or Ca_v_1.2 and CaMKK2 (Fig. 2*D*) was detected in freshly isolated mesenteric artery myocytes from WT mice. This colocalization ratio was reduced significantly in cav1-KO mice. We recognized, however, that optical resolution (200 nm) is much larger than the distance between single molecules. Therefore, we next utilized proximity ligation assays (PLA) to look for selective molecular coupling at a distance less than 40 nm. PLA signals derived from cav1-CaMKK2 and Ca_v_1.2-CaMKK2 were observed in freshly isolated mesenteric artery myocytes, but this was significantly reduced in those from cav1-KO mice (Fig. 2*E*). PLA analysis also revealed direct coupling between Ca_v_1.2 and cav1 in these mesenteric artery myocytes (*SI Appendix*, Fig. S4).

**Fig. 3.**
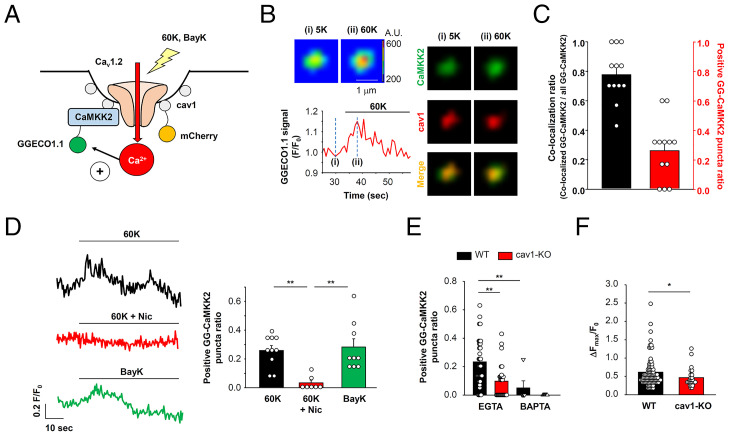
Ca^2+^ signal from Ca_v_1.2 directly activates CaMKK2 within caveolae. (*A*) A diagram of the experimental design is shown. Primary vascular myocytes were induced to express CaMKK2 labeled with GGECO1.1 at the N terminus (GG-CaMKK2). In *B* and *C*, myocytes additionally expressed cav1 tagged with mCherry (mCh-cav1). Ca^2+^ signal from Ca_v_1.2 to CaMKK2 within the same complex were monitored via changes in GGECO1.1 fluorescence in the presence of the “slow” Ca^2+^ chelator, 30 μM EGTA. (*B*) Primary myocytes expressing GG-CaMKK2 and mCh-cav1 were visualized using a TIRF microscope. Representative images of GG-CaMKK2 puncta at rest (*i*: 5 mM K^+^) and depolarization phase (*ii*: 60 mM K^+^) are shown, *Upper Left*. A trace of the GGECO1.1 signal (*F*/*F*_0_) is shown, *Bottom Left*. GG-CaMKK2 (green) and mCh-cav1 (red) puncta at the point of *i* and *ii* are shown, *Right*. Yellow signal in merged images denotes a molecular complex of GG-CaMKK2 and mCh-cav1. (*C*) The ratio of the colocalized (GG-CaMKK2+mCh-cav1) puncta number to the total GG-CaMKK2 puncta number is compared (left column). Among the colocalized puncta, the ratio of GG-CaMKK2 puncta reacting to 60 mM K^+^ solution is shown in the right column (positive GG-CaMKK2 puncta ratio). The positive puncta were determined as those whose maximal increase in fluorescence intensity (*F*_max_) due to the depolarization stimulus was higher than 5× SD of the baseline signal. Data were obtained from 12 cells. (*D*) Primary myocytes expressing GG-CaMKK2 were stimulated with 60 mM K^+^ solution with or without 10 μM Nic, or 10 μM BayK8644. The number of GG-CaMKK2 puncta reacting to each stimulus were counted and the ratio to total GG-CaMKK2 puncta within TIRF footprints was calculated (7 to 10 cells). (*E*) Primary myocytes were preincubated with EGTA-AM or BAPTA-AM, and the ratio of GG-CaMKK2 reacting to 60 mM K^+^ solution was compared between WT and cav1-KO myocytes (6 to 32 cells). (*F*) Increase in fluorescence intensity (Δ*F*_max_/*F*_0_) of positive GG-CaMKK2 puncta in WT (91 puncta from 32 cells) and cav1-KO (24 puncta from 27 cells) is compared. Statistical analysis was performed using one-way ANOVA followed by Tukey test (*D*) and two-tailed *t* test (*E* and *F*) (*P < 0.05, **P < 0.01).

Datasets from immunostaining experiments cannot prove that Ca^2+^ influx through Ca_v_1.2 directly activates CaMKK2 within the same complexes. To address this point, primary vascular myocytes were transfected with CaMKK2 that had been labeled with the genetically encoded Ca^2+^ indicator GGECO1.1 (30) (GG-CaMKK2) and mCherry-tagged cav1 (mCh-cav1) (Fig. 3*A*). These preparations were treated with EGTA-AM (30 μM) to selectively detect a reaction of GG-CaMKK2 with Ca^2+^ influx through directly coupled Ca_v_1.2. Membrane depolarization by 60 mM K^+^ solution caused an increase in the GG-CaMKK2 signal that colocalized with mCh-cav1 signals in vascular myocytes (Fig. 3*B*). The colocalization ratio of GG-CaMKK2 puncta with mCh-cav1 puncta was 0.78 ± 0.05 (12 cells) and the ratio of GG-CaMKK2 puncta that responded to a depolarization stimulus was 0.26 ± 0.06 (Fig. 3*C*). The increase in GG-CaMKK2 signal in the presence of a 60 mM K^+^ solution was reduced by the specific Ca_v_1.2 inhibitor, Nic, while BayK8644, a Ca_v_1.2 activator, induced a GG-CaMKK2 signal increase (Fig. 3*D*). These results suggest that CaMKK2 localized within caveolae is directly activated by Ca^2+^ influx through Ca_v_1.2 channels. The EGTA-resistant GG-CaMKK2 signal increase was eliminated by BAPTA (30 μM) in these WT vascular myocytes (Fig. 3*E*), a finding consistent with the presence of a functional Ca^2+^ signaling microdomain. In the case of cav1-KO vascular myocytes treated with EGTA-AM, the ratio of GG-CaMKK2 puncta that responded to a depolarization stimulus and Δ*F*_max_/*F*_0_ were decreased compared to WT (Fig. 3 *E* and *F*), although the amount of [Ca^2+^]_i_ elevation evoked by 60 mM K^+^ solution was similar between WT and cav1-KO primary vascular myocytes (*SI Appendix*, Fig. S3 *B* and *C*). These results also strongly suggest that caveolae compartmentalize Ca_v_1.2 and CaMKK2 and promote direct signal transmission from Ca_v_1.2 to CaMKK2 in vascular myocytes.

We also noted that signaling from Ca_v_1.2 to GG-CaMKK2 could be detected in some cav1-KO primary myocytes even in the presence of the slow chelator EGTA. It has been reported that cav3, the striated muscle-specific isoform of caveolin, is also expressed in smooth muscle tissues from cav1-KO mice, even after caveolae are disrupted (31). PLA analysis showed the direct interaction of cav3 with both Ca_v_1.2 and CaMKK2 in cav1-KO myocytes (*SI Appendix*, Fig. S5). Therefore, cav3 may partially support the physical interaction between Ca_v_1.2 and CaMKK2 independently of cav1 or caveolae.

### CaMKK2 Phosphorylates CaMK1a at Caveolae and Triggers the Movement of CaMK1a to the Nucleus.

**Fig. 4.**
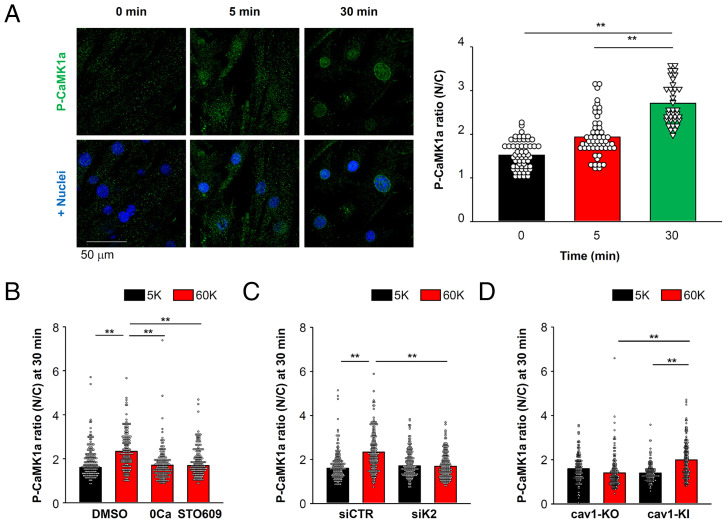
CaMK1a activation and its intranuclear localization depend on both CaMKK2 and caveolae. (*A*) Confocal images of P-CaMK1a and nuclei in vascular myocytes taken 0, 5, and 30 min after depolarization with 60 mM K^+^ solution (*Left*). The P-CaMK1a fluorescence ratio (N/C, nucleus/cytosol) at each time point are compared (*Right*). Data were obtained from 58, 54, and 36 cells from 6 confocal image sections (215 μm × 215 μm) per group at 0, 5, and 30 min, respectively. (*B*) Vascular myocytes were challenged with 60 mM K^+^ solution containing 0 [Ca^2+^]_o_ (0 Ca) or a CaMKK2 inhibitor STO609 for 30 min, and the N/C ratio of P-CaMK1a intensity was compared (162 to 171 cells per group). Before a depolarizing stimulus, myocytes were pretreated with 5 mM K^+^ solution containing 0 [Ca^2+^]_o_ or STO609 for 30 min. (*C*) The effects of CaMKK2 knockdown on 60 mM K^+^-induced CaMK1a phosphorylation (200 to 204 cells per group). (*D*) The effects of cav1-KI on 60 mM K^+^-induced CaMK1a phosphorylation (169 to 187 cells per group). In *B* to *D*, myocytes obtained from nine confocal image sections were analyzed for each group. Statistical analysis was performed using one-way (*A* and B) or two-way (*C* and *D*) ANOVA followed by Tukey test (**P < 0.01).

As illustrated in Fig. 1, CaMK1a is thought to be a downstream target of CaMKK2 that can mediate CREB phosphorylation in vascular myocytes (23). Maximum activation of CaMK1a requires both Ca^2+^/CaM binding and phosphorylation at the Thr177 site by CaMKK (23). Therefore, spatial and temporal changes in intracellular localization of phosphorylated CaMK1a were examined using confocal microscopy. Membrane depolarization by 60 mM K^+^ solution for 30 min caused CaMK1a phosphorylation in the nucleus (Fig. 4*A*). P-CaMK1a levels were increased in both cytosol and nucleus 5 min after depolarization, but P-CaMK1a signals in the nucleus were further increased compared to the cytosol at 30 min after stimulation. Accumulation of phosphorylated CaMK1a in the nucleus was inhibited by removal of extracellular Ca^2+^ or STO609 application (Fig. 4*B*). CaMKK2 knockdown also reduced CaMK1a phosphorylation (Fig. 4*C*). In addition, although intranuclear phosphorylated CaMK1a signals were not increased by 60 mM K^+^ solution in primary vascular myocytes of cav1-KO mice, knockin (KI) of cav1 resulted in reestablishment of CaMK1a phosphorylation (Fig. 4*D*). These results are consistent with a signaling pathway wherein phosphorylation of CaMK1a and its intranuclear localization require both CaMKK2 and caveolae.

**Fig. 5.**
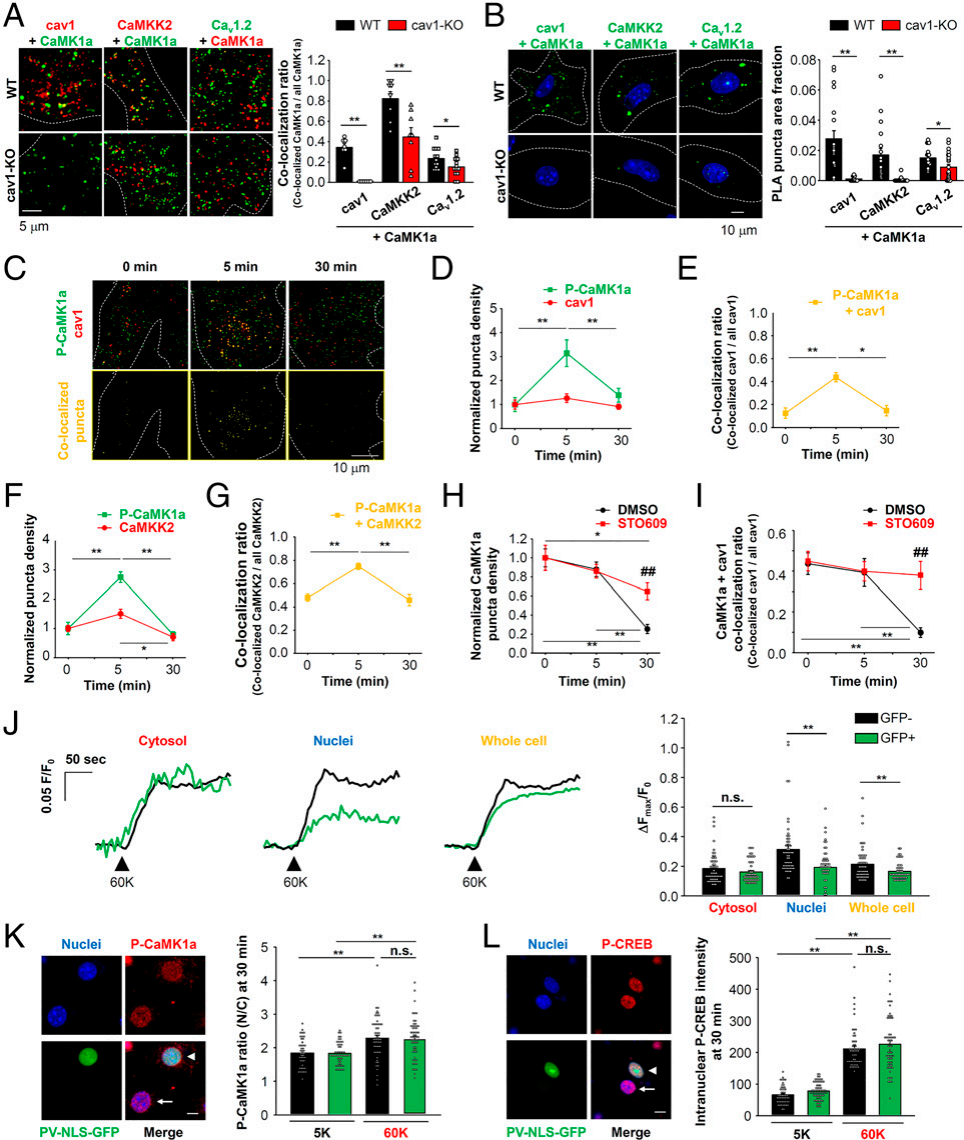
Caveolae promote a molecular complex of Ca_v_1.2/CaMK1a/CaMKK2 and full activation of CaMK1a shortly after a depolarizing stimulus. (*A*) Localization of cav1 and CaMK1a (*Left*), CaMKK2 and CaMK1a (*Center*), Ca_v_1.2 and CaMK1a (*Right*) on the plasma membrane in primary vascular myocytes was visualized using a TIRF microscope. The ratio of the colocalized puncta number to the total CaMK1a puncta number was calculated. Dashed lines indicate TIRF footprints, Data were obtained from 6 to 13 cells. (*B*) PLA analysis was performed to visualize molecular complex formation of CaMK1a with cav1, CaMKK2, and Ca_v_1.2 in primary myocytes (*Left*). PLA puncta area was normalized to the whole-cell area indicated by dashed lines (*Right*). Data were obtained from 15 to 23 cells per group. (*C*) Spatial and temporal relationships between cav1 and CaMK1a phosphorylation analyzed using a TIRF microscope. Vascular myocytes expressing mCh-cav1 were depolarized for 5 min or 30 min, then fixed and stained with P-CaMK1a antibody. P-CaMK1a, mCh-cav1, and colocalized puncta are shown in green, red, and yellow, respectively. Dashed lines indicate TIRF footprints. (*D*) The normalized density of P-CaMK1a and mCh-cav1 puncta within the TIRF footprints was plotted against depolarization period. Pooled data were obtained from 10 (0 and 5 min) and 11 (30 min) cells. (*E*) The ratio of the colocalized puncta number to the total mCh-cav1 puncta number was calculated at each time point. (*F*) Spatial and temporal relation between CaMKK2 and P-CaMK1a was analyzed. The normalized density of P-CaMK1a and CaMKK2 puncta within the TIRF footprints was plotted against the duration of the depolarization stimulus. Pooled data were obtained from 5 (0 and 30 min) and 12 (5 min) cells. (*G*) The ratio of the colocalized puncta (P-CaMK1a+CaMKK2) number to the total CaMKK2 puncta number at each time point. (*H*) The normalized CaMK1a signal density within the TIRF footprints was counted. Cells were treated with DMSO or 10 μM STO609 (7 to 11 cells per group). (*I*) The colocalization ratio between CaMK1a and cav1 was plotted. Myocytes were treated with DMSO or STO609 (7 to 11 cells per group). (*J*) Changes in Ca^2+^ levels in cytosol, nuclei, and whole cell area of primary myocytes transfected with PV-NLS-GFP were recorded using CaSiR/AM. Cells obtained from four confocal image sections (635 μm × 635 μm) were divided to two groups: GFP^−^ (black, 56 cells) and GFP^+^ (green, 50 cells) cells. Myocytes were stimulated with 60 mM K^+^ solution and peak intensity (Δ*F*_max_) normalized to basal level (*F*_0_) is compared. (*K*) The P-CaMK1a fluorescence ratio (N/C) of myocytes expressing PV-NLS-GFP (indicated by an arrowhead) or not (indicated by an arrow) is compared (41 to 57 cells). Cells were incubated with 5 mM or 60 mM K^+^ solution for 30 min. Data were obtained from 5 (5K) and 6 (60K) confocal image sections (215 μm × 215 μm). (Scale bar, 10 μm.) (*L*) The intranuclear P-CREB intensity of myocytes expressing PV-NLS-GFP (arrowhead) or not (arrow) is compared (50 to 65 cells). Data were obtained from 5 (5K) and 8 (60K) confocal image sections (215 μm × 215 μm). (Scale bar, 10 μm.) Statistical analysis was performed using two-tailed *t* test (*A*, *B*, and *J*) and one-way (*D–G*) or two-way (*H*, *I*, *K*, and *L*) ANOVA followed by Tukey test (*P < 0.05, **P < 0.01; ^##^*P* < 0.01 vs. DMSO at 30 min).

Accordingly, our working hypothesis was that CaMK1a is localized within caveolae where it forms molecular complexes with Ca_v_1.2 and CaMKK2 and can be phosphorylated by CaMKK2. TIRF imaging data revealed that molecular coupling among CaMK1a with cav1, CaMKK2, and Ca_v_1.2, and the colocalization ratio was significantly decreased in vascular myocytes from cav1-KO mice (Fig. 5*A*). PLA analysis further strengthened this possibility in both primary myocytes (Fig. 5*B*) and freshly isolated mesenteric artery myocytes (*SI Appendix*, Fig. S6). These results are consistent with cav1 having the ability to initiate molecular complexes containing Ca_v_1.2, CaMKK2, and CaMK1a in arterial myocytes. As shown in Fig. 5*C*, CaMK1a phosphorylation was enhanced 5 min after membrane depolarization at the plasma membrane (normalized P-CaMK1a puncta density: 1.0 ± 0.3 and 3.1 ± 0.6 at 0 and 5 min, respectively) (Fig. 5*D*) and these P-CaMK1a signals were convincingly colocalized with cav1 puncta (colocalization ratio: 0.12 ± 0.05 and 0.44 ± 0.04 at 0 and 5 min, respectively) (Fig. 5*E*). However, the normalized P-CaMK1a puncta density and colocalization ratios with cav1 in the plasma membrane were decreased at 30 min (1.4 ± 0.3 in Fig. 5*D* and 0.15 ± 0.05 in Fig. 5*E*). In contrast, the normalized cav1 puncta density was stable during the entire measurement time period (1.0 ± 0.2, 1.3 ± 0.2, and 0.9 ± 0.1 at 0, 5, and 30 min, respectively) (Fig. 5*D*). Similar time and spatial changes were observed in the case of P-CaMK1a and CaMKK2, although a small portion of CaMKK2 also moved to the cytosol at 30 min (Fig. 5 *F* and *G*). These findings reveal that CaMK1a phosphorylation occurred in the area where cav1 and CaMKK2 were located and that P-CaMK1a translocated from caveolae to the nucleus.

To gain further insight into these signaling component dynamics, changes in the number of CaMK1a puncta in the plasma membrane were counted with or without STO609, a CaMKK2 inhibitor. Normalized CaMK1a puncta density in the plasma membrane was unchanged at 5 min after depolarization, but deceased at 30 min. This decrease in the normalized CaMK1a puncta density was reversed by the treatment with STO609 (Fig. 5*H*). Colocalization ratios between CaMK1a and cav1 also decreased 30 min after depolarization and this was blocked by STO609 pretreatment (Fig. 5*I*). This pattern of results strongly suggests that CaMK1a can be phosphorylated by CaMKK2 within caveolae soon after depolarization, and that it is then moved to nuclei, resulting in CREB phosphorylation.

To determine the importance of nuclear Ca^2+^ levels, we overexpressed the nuclear-localized parvalbumin-based Ca^2+^ buffer protein (PV-NLS-GFP) (32). Overexpression of PV-NLS-GFP selectively reduced an increase in nuclear Ca^2+^ levels after depolarizing stimuli without any effects on cytosolic Ca^2+^ levels (Fig. 5*J*). However, PV-NLS-GFP suppressed neither P-CaMK1a translocation to the nucleus (Fig. 5*K*) nor intranuclear P-CREB accumulation (Fig. 5*L*). These results indicate that CaMK1a is phosphorylated by CaMKK2 at the cell surface, before possible translocation to the nucleus and subsequent CREB phosphorylation.

### Depolarization-Induced E-T Coupling Up-Regulates Genes Related to Chemotaxis and Inflammation.

**Fig. 6.**
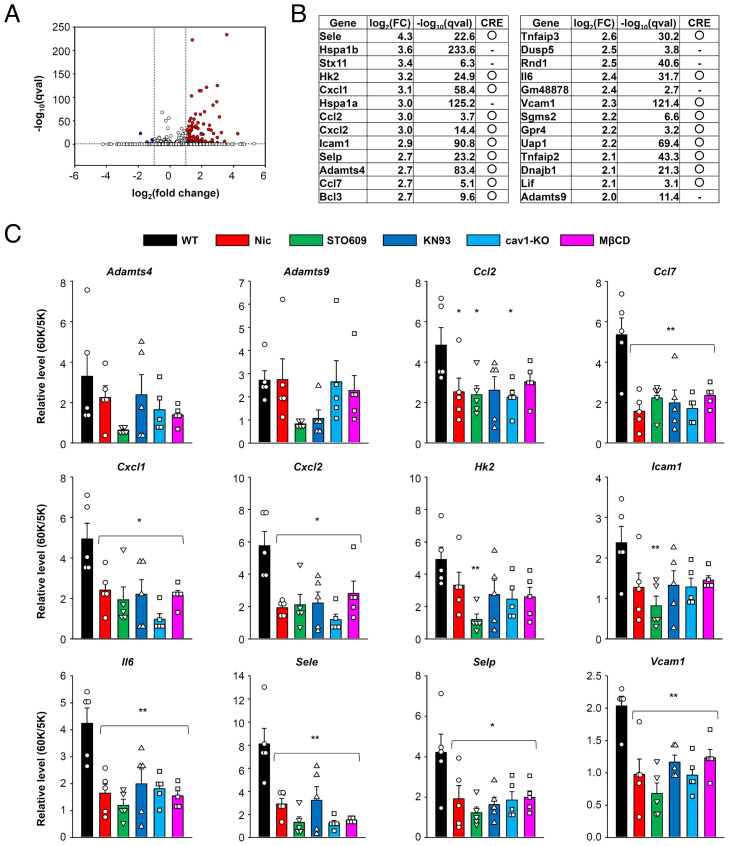
Proinflammatory genes are induced by E-T coupling in mesenteric artery. (*A*) DEG analysis was performed using mesenteric arteries treated with 5 mM or 60 mM K^+^ solution for 60 min. Detected genes are shown in volcano plots. Red and blue dots denote up-regulated (138 genes) and down-regulated (5 genes) genes, respectively. (*B*) Top 26 up-regulated genes with log_2_(fold-change) values more than 2 are listed. Genes predicted to have CRE motif are indicated in the right columns. (*C*) Gene transcription after sustained depolarization was confirmed by qPCR. Effects of Nic, STO609, KN93, cav1-KO, and MβCD were also examined. Data were obtained five mice per group. Statistical analysis was performed using one-way ANOVA followed by Dunnett test (*P < 0.05, **P < 0.01 vs. WT).

To relate these molecular signaling findings to gene transcription, genes induced by membrane depolarization were analyzed using RNA-sequencing (RNA-seq). Mesenteric artery preparations were perfused with either 5 mM K^+^ (control) or 60 mM K^+^ (depolarizing) solutions for 1 h, after which induced genes were compared. This differential expression gene (DEG) analysis identified 138 up-regulated and 5 down-regulated genes (Fig. 6*A* and *SI Appendix*, Table S1). Up-regulated genes that had log_2_(fold-change) values more than 2 are listed in Fig. 6*B*. We examined whether these genes are known targets of CREB, using the CREB Target Gene Database (33). More than a half of genes (18 of 26) are predicted to contain classic or nonclassic CRE domains and to be CREB targets. Importantly, many of up-regulated genes are related to chemotaxis (*Cxcl1*, *Cxcl2*, *Ccl2*, *Ccl7*), leukocyte adhesion (*Sele*, *Selp*, *Icam1*, *Vcam1*), degradation of extracellular matrix (*Adamts4* and *Adamts9*), or inflammation (*Il6*). These up-regulated genes were further analyzed using qPCR (Fig. 6*C*). Importantly, the observed up-regulation of some genes—such as *Cxcl1*, *Cxcl2*, *Ccl2*, *Ccl7*, *Sele*, *Selp*, *Il6*, and *Vcam1*—was reversed by inhibitors of Ca_v_1.2, CaMKK, CaMK, and caveolar disruption.

Next, we sought evidence that the observed changes occurred predominantly in the smooth muscle cells. We acknowledge the possibility that endothelial cells also participate in depolarization-induced gene transcription. Accordingly, the endothelium was physically removed by perfusing 0.2% sodium deoxycholate (34) (*SI Appendix*, Fig. S7 *A* and *B*). The perfusion of sodium deoxycholate also reduced acetylcholine-induced vasodilation (*SI Appendix*, Fig. S7*C*). In the endothelium denuded arteries, depolarization-induced gene induction was comparable to those seen with control arteries (*SI Appendix*, Fig. S7*D*). These results strongly suggest that the observed gene induction in this study mainly occurs in vascular myocytes, rather than endothelial cells.

In combination, these results strongly indicated that membrane depolarization causes E-T coupling based on a Ca_v_1.2-CaMKK2-CaMK1a-CREB pathway and induces leukocyte accumulation.

### High-Pressure Evoked E-T Coupling, Macrophage Accumulation, and Vascular Remodeling.

It has previously been reported that accumulation of leukocytes (such as neutrophils and monocyte/macrophages) can initiate vascular remodeling (16–18). It is well known that circumferential stretch produced by an increase in intraluminal pressure results in significant depolarization of the arterial membrane potential (35). Therefore, we tested the possibility that Ca_v_1.2 activation by increased circumferential stretch in mesenteric artery can induce: 1) E-T coupling, 2) leukocyte accumulation, and 3) vascular remodeling using our ex vivo and in vivo models. First, we examined whether increased perfusion pressure can cause CREB phosphorylation. Using the ex vivo model, high pressure (80 mmHg) increased the P-CREB (+) cell ratio, and this change was blocked by Nic, KN93, or STO609 (*SI Appendix*, Fig. S8 *A* and *B*). In contrast, physiological pressure (40 mmHg) did not increase this P-CREB (+) cell ratio; that is, the ratio values were approximately equal to those of mesenteric arteries that had been rapidly fixed with ice cold 4% PFA, or incubated with no transluminal pressure gradient (0 mmHg). When the same elevated pressure maneuver was also applied to mesenteric arteries from cav1-KO and MβCD-treated tissues, the P-CREB (+) cell rate was not significantly changed by the stimulus (*SI Appendix*, Fig. S8*C*).

**Fig. 7.**
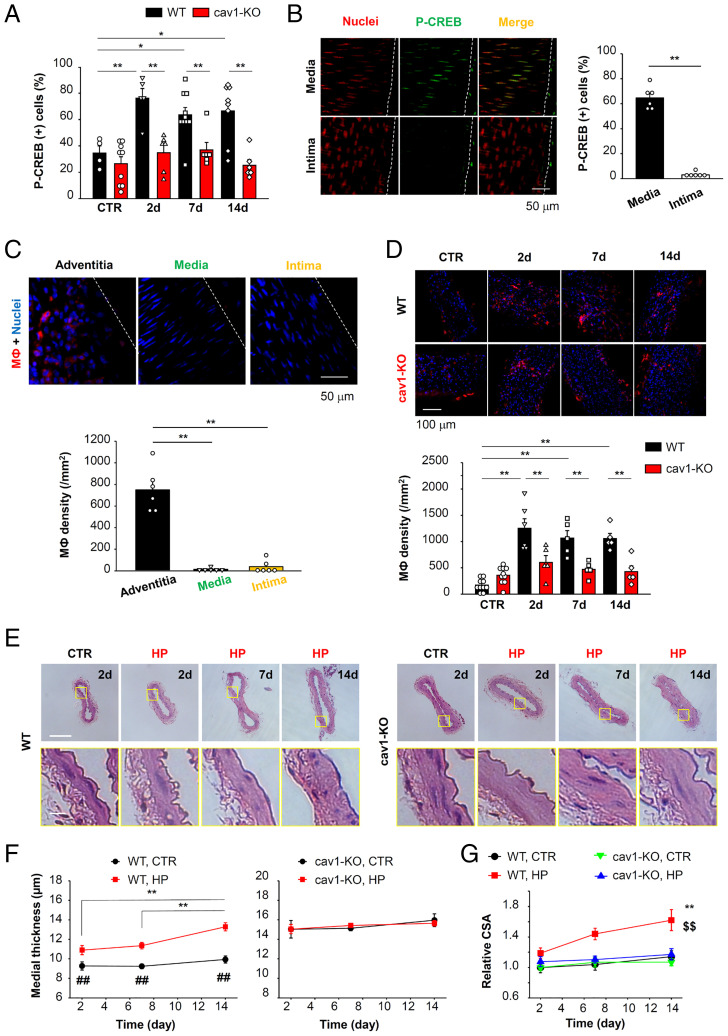
Pressure loading of mesenteric arteries results in vascular remodeling which can be reversed by cav1-KO. Elevated transluminal pressure (high pressure, HP) was applied to a second-order mesenteric artery in vivo, and tissues were sampled 2, 7, and 14 d after ligation surgery. (*A*) CREB phosphorylation was evaluated by immunostaining (4 to 10 arteries) in WT and cav1-KO. *P < 0.05, **P < 0.01. (*B*) CREB phosphorylation in endothelial cells was examined 2 d after ligation in WT (six arteries per group). **P < 0.01. (*C*) F4/80 (+) macrophages (MФ, red) accumulated in adventitia, media, and intima of WT arteries 2 d after ligation visualized using a confocal microscope (six arteries). Nuclei were stained with Hoechst (blue). **P < 0.01. (*D*) Accumulation of MФ in adventitia was analyzed in WT and cav1-KO mice (5 to 10 arteries). For each artery, three image sections (215 μm × 215 μm in *C* and 635 μm × 635 μm in *D*) were acquired and the calculated MФ density values were averaged. **P < 0.01. (*E*) Histological analysis performed using H&E-stained samples. (Scale bars, 100 μm [*Upper*] and 10 μm [*Lower*].) (*F*) Media thickness was compared between WT and cav1-KO mice. Pooled data were collected from four to seven arteries. **P < 0.01; ^##^*P* < 0.01 vs. WT HP at the same day. (*G*) Relative CSA of tunica media was compared. **P < 0.01 vs. WT CTR; ^$$^*P* < 0.01 vs. cav1-KO HP. Statistical analysis was performed using one-way (*C*) or two-way (*A*, *D*, *F*, and *G*) ANOVA followed by Tukey test and two-tailed *t* test (*B*).

In the next series of experiments, high pressure was applied to a second-order mesenteric artery in vivo by ligation of two neighboring second-order mesenteric arteries. Samples were obtained 2, 7, and 14 d after this surgery (16). Control data were obtained from second-order mesenteric arteries located along the intestine, but remote from the ligated arteries. The P-CREB (+) myocyte ratio was increased in arteries exposed to high pressure at 2, 7, and 14 d after this ligation procedure in WT mice (Fig. 7*A*). In contrast, P-CREB was not detected in endothelial cells at 2 d after ligation (Fig. 7*B*). We also examined by immunostaining whether macrophage counts increased in the stimulated arteries (Fig. 7 *C* and *D*). Increased numbers of F4/80 (+) macrophages were detected within the adventitia but not the endothelium (Fig. 7*C*) 2 d after the ligation in WT mice. These changes were maintained for at least 14 d (Fig. 7*D*). Histological analysis with H&E staining revealed that the high-pressure maneuver caused remodeling of the vascular wall (Fig. 7*E*). Specifically, the thickness (Fig. 7*F*) and cross-sectional area (CSA) (Fig. 7*G* and *SI Appendix*, Fig. S9*A*) of the tunica media were increased 7 d after ligation in WT mice. In contrast, none of the changes in the P-CREB (+) cell ratio (Fig. 7*A*), macrophage accumulation (Fig. 7*D*), increases in media thickness (Fig. 7*F*), and CSA (Fig. 7*G*) were observed in cav1-KO mice.

**Fig. 8.**
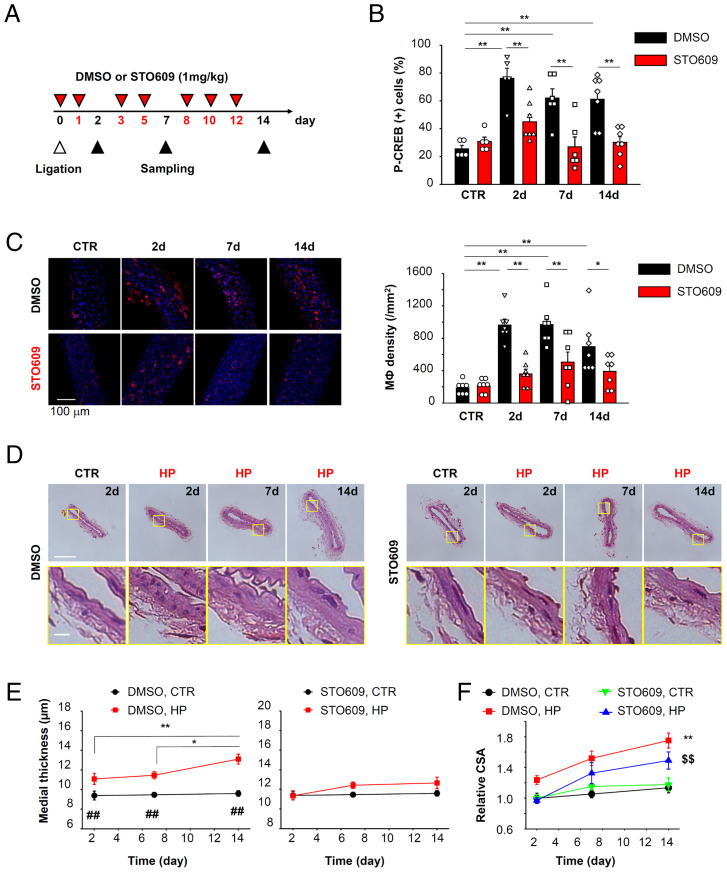
Vascular remodeling due to pressure loading can be inhibited by the CaMKK2 inhibitor STO609. (*A*) Mesenteric artery ligation was applied to WT mice and either DMSO or STO609 (1 mg/kg) was administered intraperitoneally as indicated. (*B*) CREB phosphorylation was evaluated by immunostaining. Data were obtained from five to seven arteries per group. **P < 0.01. (*C*) F4/80 (+) macrophage (MФ) density at adventitia was compared between mice treated with DMSO and STO609. Data were obtained from seven arteries per group. *P < 0.05, **P < 0.01. (*D*) Histological analysis performed using H&E-stained samples. (Scale bars, 100 μm [*Upper*] and 10 μm [*Lower*]). (*E*) Medial thickness was measured and compared between DMSO and STO609 groups. Data were collected from five to six arteries. *P < 0.05, **P < 0.01; ^##^*P* < 0.01 vs. DMSO HP at the same day. (*F*) Relative CSA of the tunica media was compared. **P < 0.01 vs. DMSO CTR; ^$$^*P* < 0.01 vs. DMSO HP. Statistical analysis was performed using two-way ANOVA followed by Tukey test.

Finally, we examined the effects of STO609 on high pressure-induced vascular remodeling in vivo. STO609 (1 mg/kg) was administered intraperitoneally before surgery, and also three times per week after surgery (Fig. 8*A*). STO609 reduced the P-CREB (+) cell ratio (Fig. 8*B*) and macrophage accumulation in adventitia (Fig. 8*C*) compared to the DMSO group. STO609 also inhibited medial hypertrophy (Fig. 8 *D*–*F* and *SI Appendix*, Fig. S9*B*). These results are consistent with the hypothesis that caveolae and CaMKK2 are critical for high pressure induced CREB phosphorylation, macrophage accumulation and vascular remodeling.

## Discussion

**Fig. 9.**
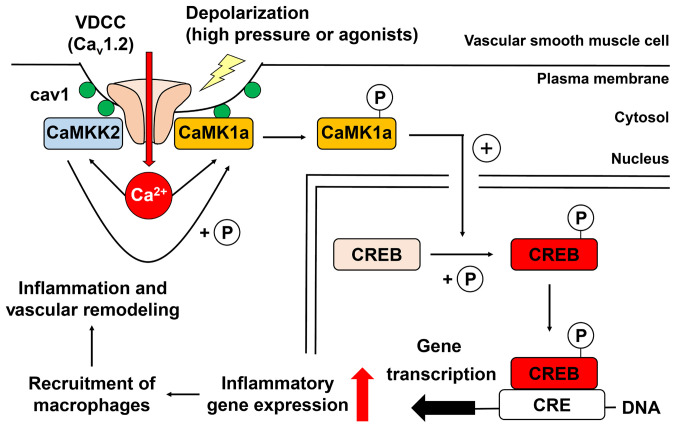
Diagram depicting molecular mechanisms that underlie E-T coupling in vascular myocytes and their roles in vascular remodeling. Caveolae can promote the formation of molecular complexes consisting of Ca_v_1.2, CaMKK2 and CaMK1a in vascular myocytes. Upon stimuli such as depolarization, pressure loading, or neurohumoral factors, Ca^2+^ influx through Ca_v_1.2 activates CaMKK2 and CaMK1a. Next, CaMKK2 phosphorylates Thr177 of CaMK1a that is localized within caveolae. This triggers translocation of P-CaMK1a to the nucleus, where P-CaMK1a phosphorylates CREB. In vascular myocytes, proinflammatory genes related to chemotaxis, leukocyte adhesion, and inflammation are then activated. The resulting chemokine and adhesion molecule gene products have the ability to enhance the recruitment of macrophages to stimulated regions, where they alter inflammation and subsequent vascular remodeling. In summary, E-T coupling, employing CaMKK2-CaMK1a-CREB pathways, can covert sustained [Ca^2+^]_i_ elevation into a selective pattern of gene transcription that can promote vascular remodeling.

Several different E-T coupling mechanisms involving calcineurin/NFATc3 (9, 10) and Rho/ROCK (11, 12) pathways and their physiological or pathophysiological roles have been proposed. Relatively long-duration membrane depolarization can cause CREB phosphorylation through E-T coupling in vascular myocytes (13, 14), but information about 1) the detailed molecular mechanisms, 2) patterns of induced genes in intact artery, and 3) physiological or pathophysiological significance is lacking. Our new data demonstrate that depolarization can selectively induce transcription of genes related to chemotaxis, leukocyte adhesion, and inflammation, secondary to activating a molecular signaling complex (Ca_v_1.2/CaMKK2/CaMK1a) that is located within caveolae of vascular myocytes. Increasing transmural pressure in the mesenteric artery by ligation in vivo significantly enhanced CREB phosphorylation, macrophage accumulation, and medial layer remodeling in cav1- and CaMKK2-dependent manner (Fig. 9).

### A Molecular Mechanism Underlying E-T Coupling in Vascular Myocytes.

In cardiac myocytes, accumulation of activated CaMK2δ in nuclear and perinuclear regions causes HDAC4 nuclear export and transcription of genes related to cardiac hypertrophy (8). In this tissue setting, Ca^2+^ is supplied to CaMK2δ by IP_3_-induced Ca^2+^ release through intranuclear IP_3_Rs, as well as CICR from the sarcoplasmic reticulum. In neurons, Ca^2+^ influx through Ca^2+^ channels in the plasma membrane activates specific downstream sensor molecules located close to the inner mouth of the channel pore. This then leads to the activation of transcription factors in the nucleus and gene induction for synaptic plasticity (6, 7). Ma et al. (22) reported that CaMK2γ_A_ transports Ca^2+^/CaM into the nucleus, resulting in CaMKK and CaMK4 activation. In addition, Cohen et al. (25) revealed that CaMK1γ, not CaMK2γ_A_, plays a similar role in parvalbumin-positive interneurons. In contrast, we find that CaMKK2/CaMK1a, but not CaMK2δ, CaMK2γ, or CaMK1γ, is responsible for E-T coupling in vascular myocytes. Thus, the molecular mechanisms found in vascular myocytes are quite different from those in cardiac myocytes and neurons.

More recently, Li et al. (36) found that in cortical neurons local Ca_v_1 activation following chronic inhibition of Na_v_ channels (>24 h) can induce translocation of CaMKK2 into the nucleus and CaMK4 activation, resulting in alternative splicing of BK channels. This has been termed “excitation-alternative splicing coupling,” and an increase in action potential duration was also observed. Our data also suggest a slight decrease in cell surface levels of CaMKK2 30 min after 60 mM K^+^ stimuli (Fig. 5*F*). The translocation of CaMKK2 may contribute to sustained activation of CaMK1 in the nucleus, resulting CREB phosphorylation and changes in gene transcription.

There is a possibility that direct protein-to-protein transfer of Ca^2+^/CaM from a protein entering the nucleus with bound Ca^2+^/CaM to a nuclear protein causes both CaMK1a activation and CREB phosphorylation, as reported in neurons (22, 25), even in the presence of the nuclear Ca^2+^ buffering protein, such as PV-NLS-GFP (Fig. 5 *J*–*L*). At this point we do not know to what extent Ca^2+^/CaM is involved in this process, and future experiments involving targeted expression of Ca^2+^/CaM binding peptides as described by Dedman and colleagues (37) may resolve this question.

The present study also revealed that cav1 promotes the formation of a Ca_v_1.2/CaMKK2/CaMK1a complex in caveolae and mediates their functional coupling in vascular myocytes (i.e., the direct activation of CaMKK2 and CaMK1a by Ca^2+^ influx through Ca_v_1.2 and phosphorylation of CaMK1a by CaMKK2). Cav1 binding motifs are well characterized (38). Notably, both CaMKK2 and CaMK1a contain canonical cav1-binding motifs (^381^YCFVFGGCPF^390^ and ^235^YEFPSPYW^242^, respectively), which is consistent with the predominant caveolar localization of these molecules. It is known that CaMK1a can phosphorylate CREB upon Ca^2+^/CaM binding and phosphorylation of Thr177 by CaMKK2 (23). Our data (Figs. 4 and 5) demonstrate that CaMK1a located within or very near caveolae is fully activated in response to a prolonged depolarizing stimulus, and then translocates into the nucleus, where it phosphorylates CREB. Cytosolic localization of CaMK1a depends on CRM1-mediated nuclear export, and is mediated through a nuclear export signal (^312^VVRHMRKLQL^321^) (39). On the other hand, translocation of CaMK1a into the nucleus depends on di-basic amino acid residues in the catalytic domain (^263^KR^264^) (39). Therefore, it is possible that Ca^2+^/CaM binding, as well as phosphorylation by CaMKK2, both result conformational changes. This may promote dissociation of CaMK1a from cav1 and trigger nuclear translocation by inhibiting CRM1-mediated nuclear export or perhaps by making the nuclear localization signal effective.

### Gene Induction in Vascular Myocytes Through E-T Coupling.

Although CREB is involved in myocyte migration (40), its roles in intact vessels and in vivo have not previously been identified. CREB is phosphorylated at Ser133 by not only CaMK but also PKA, RSK, and MSK (41), and then forms homo- or heterodimers that can bind to the CRE region on genomic DNA. This activates a wide range of genes (33). Although all CaMK isoforms can phosphorylate Ser133 of CREB, CaMK2 additionally phosphorylates Ser142 and this blocks the activation of CREB (42). These results provide a firm rationale for our finding that CaMK1a not CaMK2 phosphorylates CREB and induces gene expression in vascular myocytes.

Sustained depolarization stimuli can also up-regulate genes that promote migration of leukocytes and inflammation. CXCL1, CXCL2, CCL2, and CCL7 are chemokines that play critical roles in attracting monocytes and neutrophils. Their transcription is reported to be promoted by CREB (43, 44). CXCL1 and CCL2 are released from vascular myocytes and endothelial cells upon vascular injury (45) and mechanical stress (46, 47). Recently, it has been reported that CCL7 as well as CCL2 are produced by vascular myocytes and promote recruitment of leukocytes. This process contributes to destabilization of atherosclerotic lesions (48). Interleukin (IL)-6 is one of the major proinflammatory cytokines involved in vascular inflammatory diseases (49). Interestingly CREB-mediated signaling enhances IL-6 production (50). Adhesion molecules, such as E-selectin, P-selectin, and VCAM1, are important for leukocytes to bind to the vascular wall. P-selectin and VCAM1 are induced by vascular injury (45) and high-pressure loading (46) in vascular myocytes and endothelial cells. Importantly, up-regulation of P-selectin in vascular myocytes plays significant roles in hypoxia-induced pulmonary artery hypertension (51). Although *Vcam1* has been reported to be induced by CREB (43), it is unclear whether CREB is involved in transcription of *Sele* and *Selp*. In contrast, NF-κB is responsible to induction of *Sele* (52) and *Selp* (45), in addition to the other up-regulated genes in this study (46). In addition, NF-κB is activated by an increase in intraluminal pressure (46) and it then interacts with CREB to induce chemokine gene transcription (53). Therefore, both CREB and NF-κB may coordinate and control gene transcription in vascular wall. NFATc3 also shows intranuclear accumulation in response to increased intraluminal pressure. This occurs through both activation of the NO/PKG pathway and elevation of intracellular Ca^2+^ (54). Thus, increased intravascular pressure drives several E-T coupling pathways, and these may contribute to both physiological (maintenance of differentiated state) and pathophysiological (hypertension and vascular remodeling) responses.

Some of the depolarization-induced genes identified in this study are also expressed in endothelial cells, where depolarization as well as pressure loading could potentially influence signaling (55). However, we observed that endothelium removal did not modulate the gene induction patterns and pressure loading did not cause CREB phosphorylation in endothelial cells. Thus, it is likely that depolarizing or mechanical stimuli to arteries specifically initiate E-T coupling in vascular myocytes.

### The Significance of E-T Coupling in Vascular Remodeling.

Chemokines and adhesion molecules that are found in the inner wall of blood vessels are thought to be mainly produced by endothelial cells in response to various stimuli. These molecules are essential to promote the rolling and attachment of circulating leukocytes within intraluminal blood on the endothelium (56). This essential initiating theory is widely accepted in pathological conditions, such as atherosclerosis and neointimal formation by vascular injury. In contrast, macrophages are selectively recruited to adventitia (not the endothelium) in pressure-mediated remodeling situations (16–18). In this setting, increased pressure leads to “outward remodeling” and decreased pressure leads to “inward remodeling” (15, 16). In both cases, the migration of macrophages to the adventitia of blood vessels is an early important step for the progression of vascular remodeling. However, little is known concerning the cause of macrophage migration to the adventitia.

In the present study, data from ex vivo and in vivo models (Figs. 7 and 8 and *SI Appendix*, Fig. S8) demonstrate that maintained high pressure applied to mesenteric artery causes CREB phosphorylation in vascular myocytes, as well as macrophage accumulation in adventitia and significant medial remodeling. These important changes are induced by activation of the Ca_v_1.2/CaMKK2/CaMK1a axis. We suggest that high pressure can activate Ca_v_1.2 in vascular myocytes directly via membrane stretch (57) or indirectly by depolarization due to the activation of mechanosensitive cation channels, such as TRP (58) and Piezo1 (59), in vascular myocytes. It is also interesting to note that recent papers provide evidence for the presence/expression of the same chemokines and adhesion molecules that we detected in this study in vascular myocytes in response to a broad range of stimuli, including mechanical stress (46, 47), hypoxia (51), inflammation (60), and vascular injury (45). Thus, it is plausible that chemokines and adhesion molecules (derived from vascular myocytes) can indeed attract monocytes/macrophages from outside the vessels into the adventitia layer. Although the origin of the recruited macrophages was not addressed in this study, there are at least three possible sources. First, bone marrow-derived monocytes may infiltrate into the adventitia from the extravascular vessels due to the secretion of chemokines from vascular myocytes and subsequent differentiation into macrophages (61). Second, resident macrophages in adventitia, originally arising from embryonical or postnatal development, might proliferate or migrate to the pressurized artery (62). Third, macrophages pooled in the abdominal cavity may be drawn to the pressure-loaded tissue via the increased levels of localized chemokines (63). Further disease-specific studies are needed to address the origin of the macrophage population.

## Summary

Our results reveal components of the molecular mechanisms and signaling pathways that underlie E-T coupling in vascular smooth muscle. The common triggering signal is an increase in [Ca^2+^]_i_. This activates a spatially restricted enzyme cascade that results in transcription of genes related to chemotaxis and inflammation, and then induces macrophage migration and vascular remodeling (Fig. 9). This microdomain-mediated scheme also plays an important role in the vascular remodeling due to stress induced by pressure increments on the arterial wall.

## Materials and Methods

### Animals.

Wild-type (C57BL/6) and cav1-KO mice were obtained from Japan SLC and the Jackson Laboratory, respectively (5). All experiments were approved by the Ethics Committee of Nagoya City University (H30-P-1) and conducted in accordance with the Guide for the Care and Use of Laboratory Animals of the Japanese Pharmacological Society.

### Mesenteric Arterial Bed Preparation.

The mesenteric arterial beds were perfused with 5 mM K^+^ or 60 mM K^+^ Hanks solution using a smoothened needle inserted into the first branch of the mesenteric arteries (details in *SI Appendix*). The perfusion rate was maintained at a 0.15 mL/min to create physiological shear force (∼5 dyn/cm^2^) and to avoid endothelial cell activation using a peristaltic pump. In the present study, second- and third-order mesenteric arteries were used unless otherwise stated.

### Single-Cell Isolation and Unpassaged Culture.

Single mouse mesenteric artery myocytes were freshly isolated from second- and third-order mesenteric arteries (5). To obtain unpassaged primary vascular myocytes (29), myocytes were isolated from mouse aorta. Cells were then seeded on glass bottom dishes coated with 20 μg/mL laminin for 2 h and kept in an incubator at 37 °C with 5% CO_2_ for 5 to 7 d to expand semiconfluent. Then, medium was switched to DMEM with 0.5% FBS, 1× ITS (insulin, transferrin, and selenium) supplement and 200 μM L(+)-ascorbic acid sodium for an additional 2 to 3 d. siRNA or viral vectors were applied during this period (details in *SI Appendix*).

### Mesenteric Artery Ligation Model.

From three adjacent second-order mesenteric arteries, the first and third arteries were ligated at the distal end of the vessels using 8-0 nylon suture (16). This creates high pressure in the middle vessel. Control vessels were second-order mesenteric arteries obtained along the intestine, remote from the ligated vessels (details in *SI Appendix*).

### Transfection Using Virus Vectors.

Cav1 and CaMKK2 was labeled with mCherry or GGECO1.1 (30) at the N termini. These constructs and PV-NLS-GFP (32) were cloned into pAd/CMV/V5-DEST or BacMam pCMV-DEST vectors. Adenovirus was used for KI or overexpression experiments (Figs. 2*C*, 4*D*, and 5 *J*–*L*). On the other hand, baculovirus was utilized for TIRF imaging (Fig. 3) because of relatively low expression efficiency compared with adenovirus and high signal-to-noise ratio. Experiments were performed 48 to 72 h after infection (details in *SI Appendix*).

### TIRF Imaging.

Two-dimensional single-molecular imaging were done using a TIRF imaging system (Nikon) (5, 19). For colocalization analysis, two molecules were labeled with green or orange indicators, and corresponding fluorescence puncta having higher intensity than background levels were converted to binary images. Then, colocalized puncta were extracted by image arithmetic operations using NIS Elements software (Nikon). The ratio of the number of colocalized puncta to the total number of puncta was calculated. For GGECO imaging, fluorescence signals from GGECO1.1 are described as *F*/*F*_0_, where *F* is the sum fluorescence intensity within the regions of interest (ROI, 2 μm × 2 μm) in the TIRF area during measurements, and *F*_0_ is the baseline *F* value obtained as the average intensity of the ROI for 5 s before stimulation. GGECO1.1 puncta whose maximal increase in fluorescence intensity due to depolarization stimulus was higher than 5× SD of baseline values were accepted as positive GGECO1.1 puncta (details in *SI Appendix*).

### Confocal Imaging.

Confocal images were obtained using a laser-scanning confocal fluorescent microscope (A1R, Nikon). For P-CREB analysis, fluorescence signals from secondary antibodies binding to P-CREB primary antibodies and nuclear indicators (Hoechst or TO-PRO3) were converted into binary images. Next, fluorescence signals of nuclei colocalized with P-CREB signals were extracted by image arithmetic operations using the NIS Elements software. The ratio of the number of nuclei colocalized with P-CREB signals to the total number of nuclei was calculated. For each artery, three image sections (215 μm × 215 μm) were acquired and the calculated ratio values were averaged (details in *SI Appendix*).

### Ca^2+^ Imaging.

Intracellular Ca^2+^ imaging was performed using a confocal microscope (details in *SI Appendix*). When Ca^2+^ responses of myocytes transfected with PV-NLS-GFP were recorded, cells were loaded with 6.7 μM CaSiR/AM and Hoechst. ROIs corresponding to whole-cell regions and nuclei were created according to the fluorescence of CaSiR and Hoechst, respectively. The sum of CaSiR intensity and area corresponding to cytosol were calculated by subtracting these values of nuclei from those of whole-cell regions.

### Data Notation and Statistical Analysis.

Pooled data are shown as the mean ± SEM. The significance of differences between two groups was evaluated using the two-tailed *t* test after the application of the *F* test. Data from more than two groups were compared using one-way or two-way ANOVA followed by Tukey or Dunnett tests. In all cases, *P* < 0.05 was considered to be significant. All data were obtained from at least three independent experiments.

All details of immunostaining, ELISA, histological analysis, siRNA knockdown, real-time qPCR, RNA-seq analysis, in situ PLA, and drugs are provided in *SI Appendix*. Details are also provided of sources and reference numbers for reagents, recombinant DNA, and oligonucleotides necessary for replication in *SI Appendix*.

## Supplementary Material

Supplementary File

## Data Availability

The RNA-seq data reported in this paper have been deposited in the Gene Expression Omnibus (GEO) database, https://www.ncbi.nlm.nih.gov/geo (accession no. GSE183588). All other study data are included in the main text and *SI Appendix*.
