# Supplementary material for: A molecular complex of Cav1.2/CaMKK2/CaMK1a in caveolae is responsible for vascular remodeling via excitation–transcription coupling
Source: Proc Natl Acad Sci U S A. 2022 Apr 11;119(16):e2117435119. doi: 10.1073/pnas.2117435119 (PMC9169798; doi:10.1073/pnas.2117435119)
Supplement: Supplementary File [file pnas.2117435119.sapp.pdf]

PNAS
www.pnas.org

Supplementary Information for
A molecular complex of $Ca_V1.2$/CaMKK2/CaMK1a in caveolae is responsible for vascular remodeling via excitation-transcription coupling

Yoshiaki Suzuki*, Takumi Ozawa, Tomo Kurata, Nanami Nakajima, Gerald W. Zamponi, Wayne R. Giles, Yuji Imaizumi and Hisao Yamamura*.

*Yoshiaki Suzuki and Hisao Yamamura.
Email: yoshisuz@phar.nagoya-cu.ac.jp and yamamura@phar.nagoya-cu.ac.jp

This PDF file includes:

Supplementary Text
Figures S1 to S9
Tables S1 to S2

Supplementary Information Text

Materials and Methods

Animals

Wild type (C57BL/6) and cav1-KO mice (8-12 weeks, male) were obtained from Japan SLC and Jackson Labs, respectively (1). All experiments were approved by the Ethics Committee of Nagoya City University (H30-P-1) and conducted in accordance with the Guide for the Care and Use of Laboratory Animals of the Japanese Pharmacological Society.

Mesenteric arterial bed preparation.

The mesenteric arterial bed was removed from the intestine after banding distal arterioles. It was placed in ice cold Ca^{2+}/Mg^{2+}-free Hanks solution: (mM) 137 NaCl, 5.4 KCl, 0.39 Na_2HPO_4, 0.44 KH_2PO_4, 4.2 $NaHCO_3$, and 5.6 glucose and a smoothened needle (0.6 mm in diameter) was inserted into the first branch of the mesenteric arteries. The perfusion rate was maintained at a 0.15 ml/min to create physiological shear force (~5 dyn/cm^2) and to avoid endothelial cell activation using a peristaltic pump (MP-3B, TOKYO RIKAKIKAI). The mesenteric arterial bed preparation was perfused with Ca^{2+}/Mg^{2+}-free Hanks solution to remove blood for 15 min and then switched to standard Hanks solution additionally containing 2.2 mM $CaCl_2$ and 1.2 mM $MgCl_2$ with or without drugs. The tissue was then transferred to a dish containing the same solution and was incubated without perfusion for an additional 45 min at 37°C. The first order of mesenteric arteries was ligated at center and the distal side of the arteries were harvested for a 5 mM K^+ control. Remaining tissue connected to the needle was attached to the peristaltic pump and perfused with 60 mM K^+ Hanks solution containing 82 mM NaCl and 60 mM KCl for 30 to 60 min. In experiments in which the endothelium was removed, arteries were perfused with 0.2% sodium deoxycholate dissolved in ice cold PBS for 120 sec (34). Endothelial removal was confirmed by (i) confocal imaging, (ii) qPCR analysis of the endothelium marker, von Willebrand factor (vWF), and (iii) pressure recording. Harvested tissues were immersed in or perfused with ice cold PBS with 4% paraformaldehyde (PFA) for immunostaining. In the case of RNA or protein extraction, tissues were flash-frozen with liquid N_2 after removal of connective tissues. High (80 mmHg) or low (40 mmHg) pressure was loaded to mesenteric arteries by adjusting the flow speed using a peristaltic pump (2). Perfusion pressure was monitored using a pressure-displacement transducer (Baxter) connected to a needle. All solutions contained 100 nM tetrodotoxin (LATOXAN) to avoid the activation of autonomic nerves by 60 mM K^+ solution. In the present study, second and third-order mesenteric arteries were used unless otherwise stated.

Single cell isolation and unpassaged culture

To isolate single mouse mesenteric artery myocytes (1), second and third-order mesenteric arteries were incubated in Ca^{2+}/ Mg^{2+}-free Hanks solution containing 0.4% collagenase and 0.1% papain (MilliporeSigma) at 37°C for 40 min. After the incubation, tissues were washed in ice cold Ca^{2+}/ Mg^{2+}-free Hanks solution and treated by gentle agitation with a glass pipette. For unpassaged cultured mouse aorta myocytes (3), mouse aortas were dissected out and placed in ice-cold DMEM containing 1×penicillin/streptomycin/amphotericin B. Connective tissue was removed in sterile PBS and incubated in a DMEM solution containing 0.2% collagenase at 37°C for 45 minutes. Then the adventitia was removed in PBS and transferred to a DMEM solution containing 0.2% collagenase and 0.2% papain at 37 for 60 min to disperse myocytes. Before starting the incubation, the tissue was cut into 2-5 mm segments. The digested tissue was then centrifuged for 5 minutes at 3,000 rpm and the solution was replaced with fresh DMEM solution containing 10% FBS (Gibco). The pellet was gently resuspended, which resulted in dispersion of individual arterial myocytes. Cells were then seeded on glass bottom dishes (Matsunami) coated with 20 µg/mL laminin (MilliporeSigma) for 2 hours and kept in an incubator at 37C with 5% CO_2 for 5-7 days to expand semi-confluent. Then, medium was switched to DMEM with 0.5% FBS, 1×ITS (insulin, transferrin and selenium) supplement and 200 µM L(+)-ascorbic acid sodium for additive 2-3 days. siRNA or viral vectors were applied during this period. For experiments, cells were incubated with standard (5 mM K^+) HEPES-buffered solution: (mM) 137 NaCl, 5.9 KCl, 2.2 $CaCl_2$, 1.2 $MgCl_2$,14 glucose, and 10 HEPES or 60 mM K^+ HEPES-buffered solution: (mM) 83 NaCl, 60 KCl, 2.2 $CaCl_2$, 1.2 $MgCl_2$,14 glucose, and 10 HEPES. When the smooth muscle myocytes were stimulated with BayK8644, $CaCl_2$ concentration was 20 mM and NaCl concentration was reduced to 105 mM (4). The pH was adjusted to 7.4 with NaOH.

Mesenteric artery ligation model

WT and cav1-KO mice (male or female, 12 weeks) were anesthetized by chloral hydrate. From three adjacent second-order mesenteric arteries, the first and third arteries were ligated at the distal end of the vessels using 8-0

nylon suture (Natsume Seisakusho) (5). This creates high pressure in the middle vessel. Control vessels were second-order mesenteric arteries obtained along the intestine, remote from the ligated vessels. The mesenteric artery was isolated 2d, 7d or 14 d after surgery. To evaluate the effects of STO609 in vivo, mice were treated with STO609 (1 mg/kg) intraperitoneally on the day of surgery (at least 2 hours before surgery) and then three time per week. STO609 was dissolved in DMSO at 10 mg/mL and stored at -20°C until usage. This stock solution was diluted to 0.2 mg/mL with sterile PBS. A PBS solution containing 2% DMSO was used as a vehicle control.

Immunostaining
Mesenteric arteries, freshly isolated and primary vascular myocytes were fixed with 4% PFA in PBS. These were treated with 0.2% Triton X-100, 3% bovine serum albumin (BSA, MilliporeSigma), and anti-Phospho-CREB (Ser133) rabbit monoclonal antibody (1:300, 87G3, CST), anti-CaMKI (phospho T177) rabbit antibody (1:300, ab62215, Abcam), anti-CaMKK2 rabbit antibody (1:200, GTX115950, GeneTex), anti-CaMKK2 mouse antibody (1:200, 6/CaM Kinase, 610544, BD Biosciences), anti-cav1 rabbit antibody (1:1000, C4490, MilliporeSigma), anti-cav1 mouse antibody (4H132, SantaCruz), anti-cav3 rabbit antibody (1:500, GTX109650, GeneTex), anti-Ca_v1.2 mouse monoclonal antibody (1:200, N263/31, NeuroMab) or anti- smooth muscle actin (SMA) mouse monoclonal antibody (1:1000, 1A4, CST) at 4°C for 12 h. After washing repeatedly, the preparations were treated with Alexa 488-, Alexa 594- or Alexa 647-conjugated secondary antibodies (1:500, Molecular Probes) at room temperature for 1 h. In some experiments, cav1 antibody conjugated with HiLyte Fluor 555 (Dojindo) was used for dual staining with another rabbit antibodies. For detection of macrophages, PE conjugated anti F4/80 antibody (1:100, REA126, Miltenyi Biotec) was utilized. Nuclei were stained with TO-PRO3 (1:500, Thermo Fisher Scientific) or Hoechst (1:500, Thermo Fisher Scientific) at room temperature for 30 min. The mesenteric artery was mounted on coverslips (Matsunami) using Dako fluorescence mounting medium (Agilent Technologies). Fluorescently labeled samples were observed using a confocal or TIRF imaging system.

ELISA
The P-CREB levels in arteries were quantified using ELISA. Two types of ELISA detecting (i) phosphorylated CREB at S133 (KHO0241, Thermo Fisher Scientific) and (ii) total CREB (KHO0231, Thermo Fisher Scientific) were performed using arterial lysates according to protocols provided by the company. Second and third-order mesenteric arteries were flash-frozen in liquid N_2. The frozen samples were pulverized and proteins were extracted using a cell lysis buffer (100 μL) containing 10 mM Tris, pH 7.4, 100 mM NaCl, 1 mM EDTA, 1 mM EGTA, 1 mM NaF, 20 mM $Na_4P_2O_7$, 2 mM Na_3VO_4, 1% Triton X-100, 10% glycerol, 0.1% SDS, 0.5% deoxycholate (Thermo Fisher Scientific). Protease inhibitor cocktail (MilliporeSigma) and 1 mM PMSF (TCI) were added just prior to experiments. The lysates were sonicated briefly and centrifuged to remove debris. The supernatant was diluted to 1:20 and subjected to ELISA. Both phosphorylated and total CREB amounts of each sample were calculated from standard curves, and the relative P-CREB level to total CREB of each sample was obtained.

Histological analysis
Isolated arteries were fixed in 4% PFA, embedded in paraffin, and cut into 5-μm thick sections for routine hematoxylin-eosin (HE) staining (Biopathology Institute). Medial thickness was measured at 4 different points and averaged per sample. Image analysis was performed using NIS Elements (Nikon).

Transfection using virus vectors
Cav1 (NM_001753) and CaMKK2 (XM_006530233) was labeled with mCherry or GGECO1.1 (Addgene plasmids #32445) at the N termini. pCMV-PV-NLS-GFP was obtained from Addgene (#17300). These constructs were cloned into pENTR1A and obtained pAd/CMV/V5-DEST or BacMam pCMV-DEST vectors using Gateway system (Thermo Fisher Scientific). Adenovirus and baculovirus vector were harvested from 293A and Sf9 cells after introducing linearized pAd/CMV/V5-DEST and bacmid, respectively. Adenovirus was used for knock-in or overexpression experiments (Fig. 2C, 4D and 5J-L). On the other hand, baculovirus was utilized for TIRF imaging (Fig. 3) because of relatively low expression efficiency compared with adenovirus and high signal to noise ratio. Primary vascular myocytes at semiconfluent condition were infected for 12 hours and then washed. Experiments were performed 48-72 hours after infection.

siRNA knockdown.
Genes were knocked down using specific siRNA (NIPPON GENE). Cells were transfected with siRNA using Lipofectamine RNAiMAX Reagent (Thermo Fisher Scientific). Two siRNA (#1 and #2, 100 nM each) targeting different regions of the same gene were simultaneously transfected. Negative control siRNA was also purchased

from NIPPON GENE. All experiments were performed between 48 h and 72 h after siRNA treatment. The sequence of gene specific siRNA was as follows. siCaMKK2 #1: GGAUUGUGGUGCCUGAAAU, siCaMKK2 #2: GCGACGCCUUGCUGUCUAA, siCaMK1a #1: GAACGAGAUUGCCGUCUUA and siCaMK1a #2: CCCACCCUUUUAUGAUGAA.

Real-time qPCR.

Total RNA extraction, reverse transcription and real-time quantitative PCR were carried out as previously reported (6). The sequence of primers was listed in Table S2. For normalization of gene expression among each samples, *Ppia* and *Hprt* were utilized as endogenous housekeeping genes because these expression was similar between 5 mM and 60 mM K^+ conditions. Averaged Cq value of these genes was used for calculation.

RNA sequence analysis

RNA was extracted from samples using TRIzol and PureLink RNA spin columns (Thermo Fisher Scientific). Genomic DNA was digested using TURBO™ DNase (Thermo Fisher Scientific). Conventional RNA-Seq was performed by Novogene. The RNA-seq reads were aligned with the mouse reference genome mm9 using TopHat2 software. Prior to differential gene expression analysis, the read counts were adjusted for each sequenced library by edgeR program package through one scaling normalized factor. Differential expression analysis of two conditions was performed using the DEGSeq R package. The P values were adjusted using the Benjamini & Hochberg methods. Corrected qvalue of 0.005 and $|\log_2$ (Fold Change)$|$ of 1 were set as the threshold for significantly differential expression.

TIRF imaging

Two-dimensional single-molecular imaging were done using a TIRF imaging system (Nikon), which consists of a fluorescent microscope (Ti2; Nikon), objective lens (CFI Plan Apo TIRF 60× or 100×; Nikon), EM-CCD camera (C9100-12; Hamamatsu Photonics), and NIS Elements software (Nikon) (1, 6). For co-localization analysis, two molecules were labeled with green or orange indicators, and corresponding fluorescence puncta having higher intensity than background levels were converted to binary images. Then, co-localized puncta were extracted by image arithmetic operations using the NIS Elements software. The ratio of the number of co-localized puncta to the total number of puncta was calculated. For GGECO imaging, fluorescence signals from GGECO1.1 are described as F/F_0, where F is the sum fluorescence intensity within the regions of interest (ROI, 2 μm square) in the TIRF area during measurements, and F_0 is the baseline F value obtained as the average intensity of the ROI for 5 sec before stimulation. GGECO1.1 puncta whose maximal increase in fluorescence intensity due to depolarization stimulus was higher than 5×SD of baseline values were accepted as positive GGECO1.1 puncta. Images were collected at an interval of 800 msec for two color imaging, i.e. GG-CaMKK2 and mCh-cav1 or 100 msec for single color imaging, i.e. GG-CaMKK2 only. Immunolabeling images were collected by exposure for 100 msec. The resolution of images was 270 or 160 nm per pixel (x − y) and less than 200 nm (z). All experiments were performed at room temperature.

Confocal imaging

Confocal images were obtained using a laser scanning confocal fluorescent microscope (A1R, Nikon) equipped with a fluorescent unit (ECLIPSE Ti), objective lens (Plan Apo 60× or 20×), and NIS Elements software (Nikon). For P-CREB analysis, fluorescence signals from secondary antibodies binding to P-CREB primary antibodies and nuclear indicators (Hoechst or TO-PRO3) were converted into binary images. Next, fluorescence signals of nuclei co-localized with P-CREB signals were extracted by image arithmetic operations using the NIS Elements software. The ratio of the number of nuclei co-localized with P-CREB signals to the total number of nuclei was calculated. For each artery, three image sections (215 μm×215 μm) were acquired and the calculated ratio values were averaged.

Ca^{2+} imaging

Intracellular Ca^{2+} imaging was performed using a confocal microscope. For monitoring $[Ca^{2+}]_i$, mesenteric artery myocytes were incubated with 10 μM Fluo-4/AM for 20 min at room temperature. The excitation and emission wavelength were 488 nm and 500-530 nm, respectively. When the amplitude of $[Ca^{2+}]_i$ elevation in primary vascular myocytes of WT and cav1-KO cells were recorded, cells were loaded with 10 μM Fluo-4/AM and 10 μM Fura Red/AM for 30 min to perform ratio metric measurement. These indicators were excited at 488 nm and emission was collected at 500-530 nm (Fluo-4) and 662-737 nm (Fura-Red). F_{ratio} was calculated by dividing fluorescence intensity of Fluo-4 by that of Fura-Red. When Ca^{2+} responses of myocytes transfected with PV-NLS-GFP were recorded, cells were loaded with 6.7 μM CaSiR/AM (Goryo Chemical) and Hoechst. These indicators were excited at 640 and 405 nm and emission was collected at 662-737 nm (CaSiR) and 425-475 nm (Hoechst). ROIs

corresponding to whole cell regions and nuclei were created according to the fluorescence of CaSiR and Hoechst, respectively. The sum of CaSiR intensity and area corresponding to cytosol were calculated by subtracting these values of nuclei from those of whole cell regions.

In situ PLA

To clarify whether cav1/Ca_v1.2/CaMKK2/CaMK1a co-localizes within 40 nm in arterial myocytes, a PLA was performed using a PLA kit (Duolink, MilliporeSigma) (6). Myocytes were fixed with 4% paraformaldehyde in PBS and treated with 0.2% Triton X-100. Cells were then labeled with primary antibodies mentioned above at 4°C for 12 h. After washing repeatedly, cells were incubated in a humidified chamber at 37°C for 1 h with secondary anti-mouse PLUS and anti-rabbit MINUS PLA probes and then washed in Duolink Wash Buffer A. The preparations were incubated in ligase solution at 37°C for 30 min in a humidifier chamber and then washed repeatedly in Wash Buffer A. Samples were incubated in Amplification Polymerase solution at 37°C for 100 min in a humidifier chamber and then washed repeatedly in Duolink Wash Buffer B. Fluorescence images were observed using a confocal imaging system. When two PLAs probes were within 40 nm, positive signals (green puncta) were generated. The excitation of fluorescent puncta was illuminated at 488 nm. Negative control experiments were performed using myocytes treated with either of antibodies. PLA puncta were extracted by binary image processing and the relative area of these puncta to the whole cell area was calculated.

Data notation and statistical analysis

Pooled data are shown as the mean ± SEM. The significance of differences between two groups was evaluated using the two-tailed t test after the application of the F test. Data from more than two groups were compared using one-way or two-way analysis of variance (ANOVA) followed by Tukey or Dunnett tests. In all cases, p values <0.05 were considered to be significant. All data were obtained from at least three independent experiments.

Drugs

Drugs were purchased from FUJIFILM Wako Pure Chemical except for BayK8644 and nicardipine (MilliporeSigma), EGTA-AM (Setareh), BAPTA-AM and HEPES (Dojindo), KN93 and MβCD (TCI), and STO609 (Cayman). All hydrophobic compounds were dissolved in dimethyl sulfoxide (DMSO) at a concentration of 10-30 mM as a stock solution.

Data Availability.

The datasets generated in this study are available at https://www.ncbi.nlm.nih.gov/geo/query/acc.cgi?acc=GSE183588

Supplementary Figures

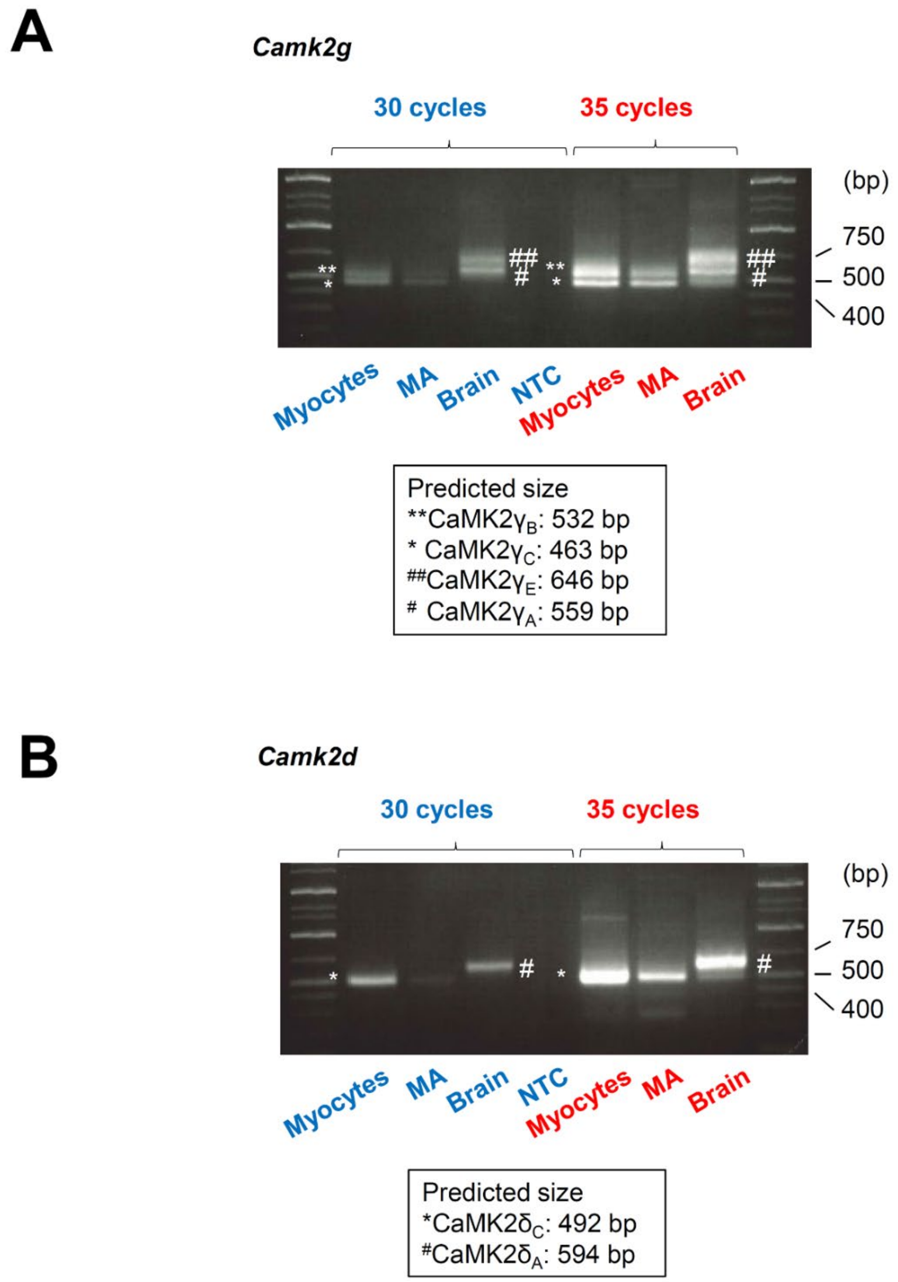

Fig. S1. Expression profiles of CaMK family genes in mesenteric artery and primary vascular myocytes.
(A and B) Splice variants of CaMK2γ (*Camk2g*, A) and CaMK2δ (*Camk2d*, B) in primary vascular myocytes, mesenteric artery (MA), and brain were identified using reverse-transcription PCR analysis. Negative control data was obtained by adding water instead of cDNA (non-template control, NTC). In both Panel (A) and (B), predicted band size for each splice variant is indicated within the box below. Representative photos from three independent experiments are shown.

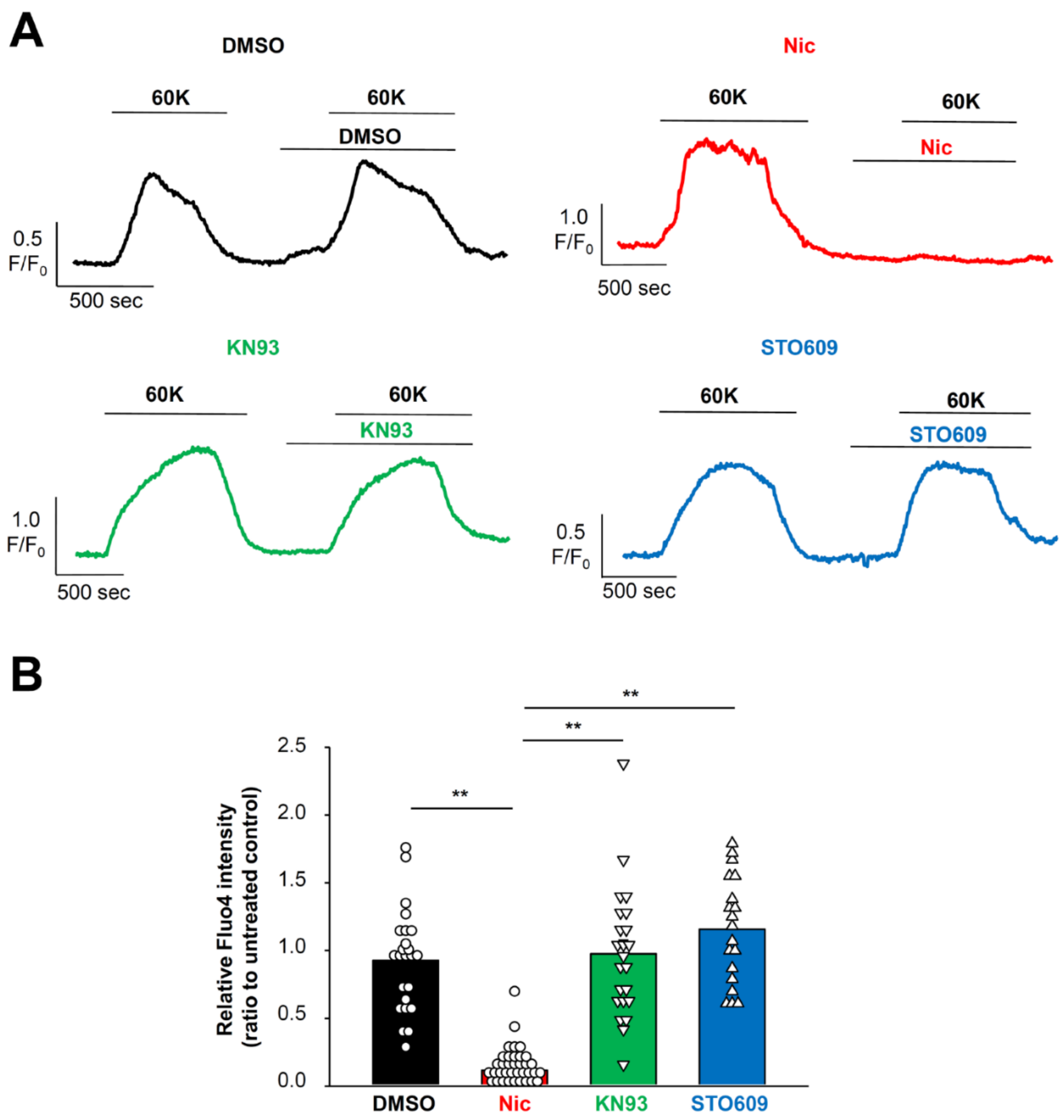

Fig. S2. Effects of CaMK inhibitors on 60 mM K^+ superfusate-induced $[Ca^{2+}]_i$ elevation by Ca^{2+} influx through voltage-dependent Ca^{2+} channels in mesenteric artery myocytes.

(A) Traces of fluorescence signals from Fluo-4/AM in mesenteric artery myocytes. Myocytes were stimulated by 60 mM K^+ solution twice, and drugs were applied at the second 60 mM K^+ stimulation. **(B)** Relative Fluo-4 intensity normalized to untreated control (the first 60 mM K^+ stimulation). Data were obtained from 19-43 cells from three independent experiments. Statistical analysis was performed using one-way ANOVA followed by Tukey test (**$p<0.01$).

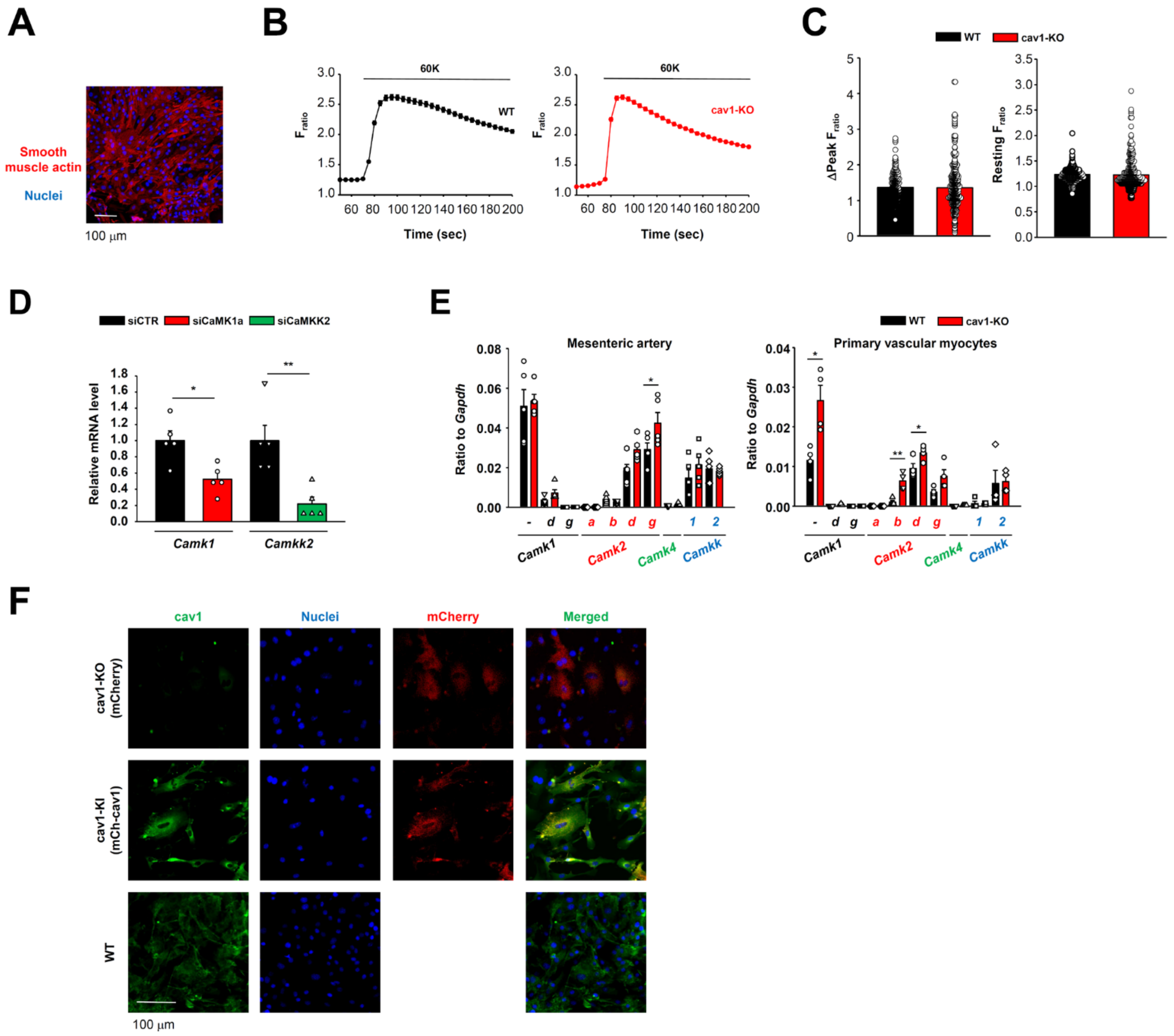

Fig. S3. Illustration and validation of essential features of unpassaged primary vascular myocytes.
(A) Unpassaged primary vascular myocytes were stained with anti-smooth muscle actin antibody. Nuclei were stained with Hoechst. Representative pictures from three independent experiments are shown. **(B)** Traces of mean and standard error of fluorescence ratio (F_{ratio}, Fluo-4/Fura-Red) in primary vascular myocytes from WT and cav1-KO mice are shown (297 and 423 cells from WT and cav1- KO mice). **(C)** Peak amplitude of F_{ratio} (left) and resting F_{ratio} (right) are compared between vascular myocytes from WT and cav1-KO mice. Data were obtained from 297 (WT) and 423 (cav1-KO) cells from four sections (635 μm×635 μm). **(D)** Knockdown efficiency by siRNA was confirmed by qPCR. Data were obtained from five independent primary (unpassaged) cultures per group. **(E)** mRNA levels of CaMK family genes in mesenteric artery (left) and primary vascular myocytes (right) are compared between WT and cav1-KO mice. Data were obtained from five mice (mesenteric artery) and four independent unpassaged cultures (primary vascular myocytes). **(F)** The cav1 expression (green) in cav1-KO myocytes expressing mCherry (cav1-KO, upper) or mCh-cav1 (cav1-KI, middle) or in WT myocytes (WT, bottom). Nuclei and mCherry are shown in blue and red, respectively. Representative pictures from three independent experiments are shown. Statistical analysis was performed using two-tailed t test (*p<0.05, **p<0.01).

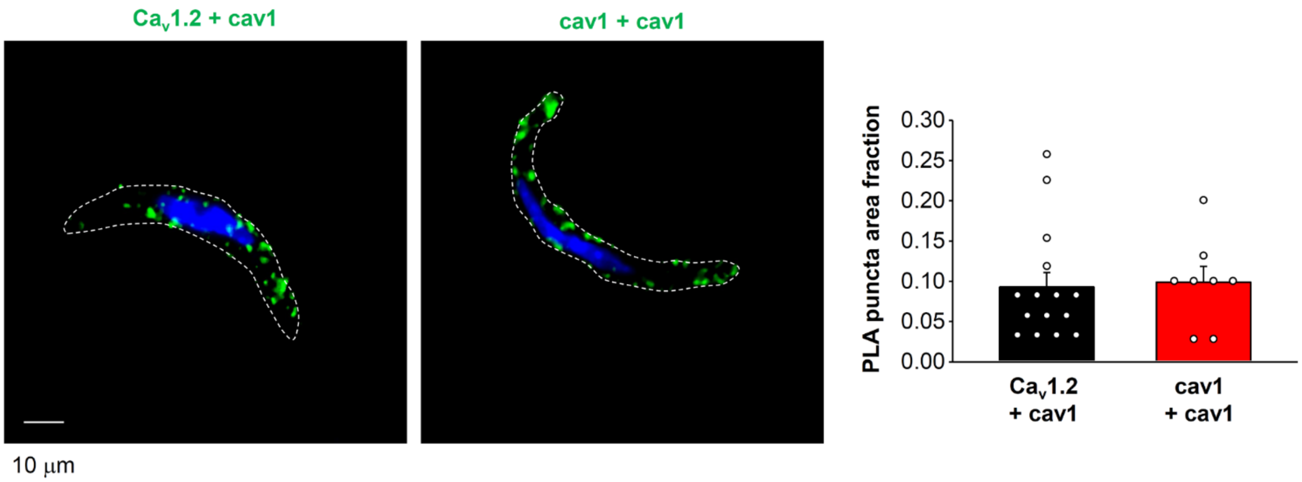

Fig. S4. Ca$_V$1.2 is coupled to cav1 in mesenteric artery myocytes.
PLA was performed to obtain evidence for direct coupling of Ca$_V$1.2 with cav1 in mesenteric artery myocytes (15 cells). Cav1 was labeled with two different antibodies from mouse and rabbit and then subjected to PLA to obtain positive control data (8 cells).

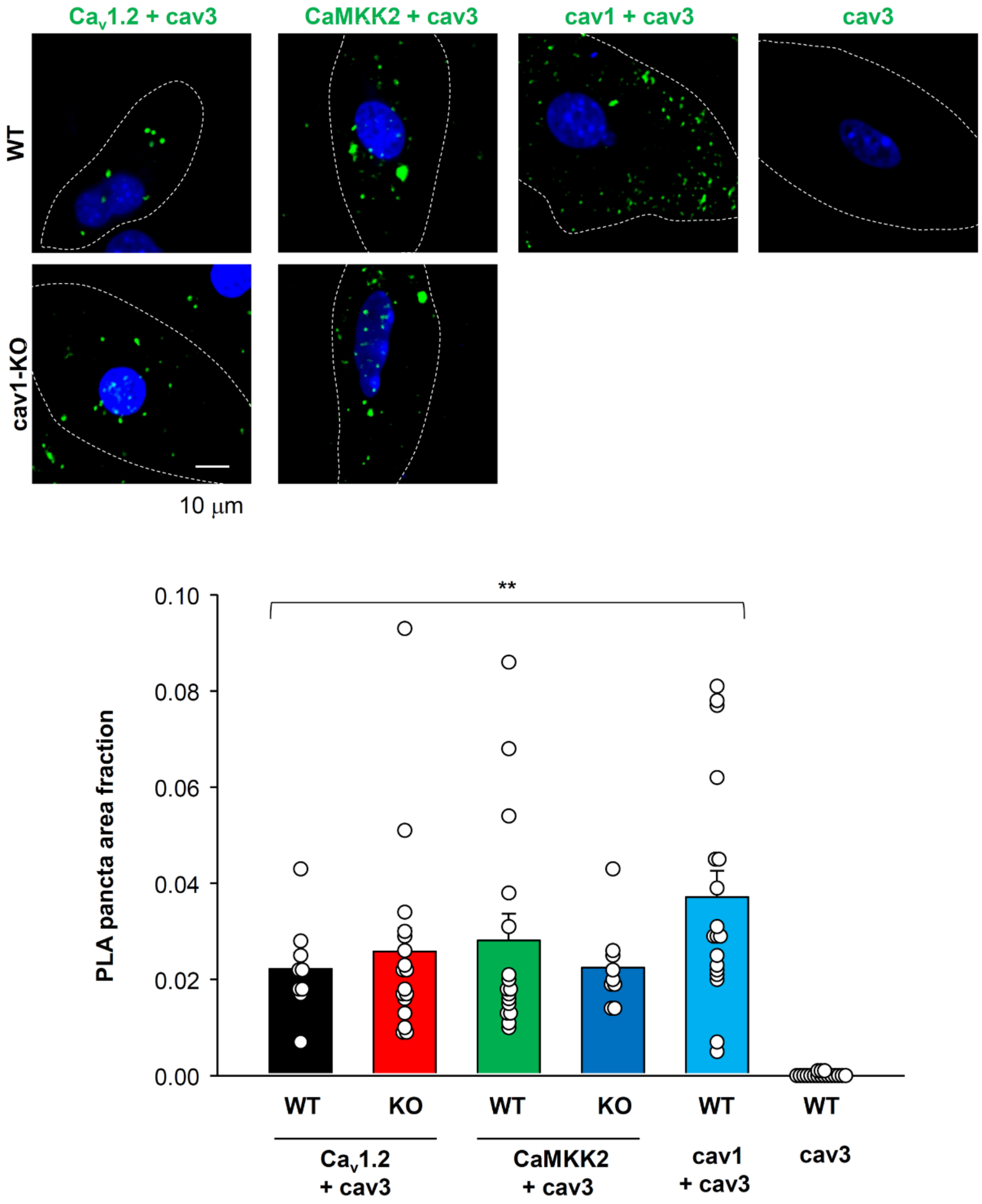

Fig. S5. Ca$_V$1.2 and CaMKK2 are coupled with cav3 in primary vascular myocytes from WT and cav1-KO mice.

PLA was performed to seek evidence for direct coupling of cav3 with Ca$_V$1.2 and CaMKK2 in primary myocytes. WT cells labeled with only the cav3 antibody were used for a negative control. Each data set was obtained from 9-18 cells. Statistical analysis was performed using one-way ANOVA followed by Tukey test (**$p<0.01$ vs. WT cav3).

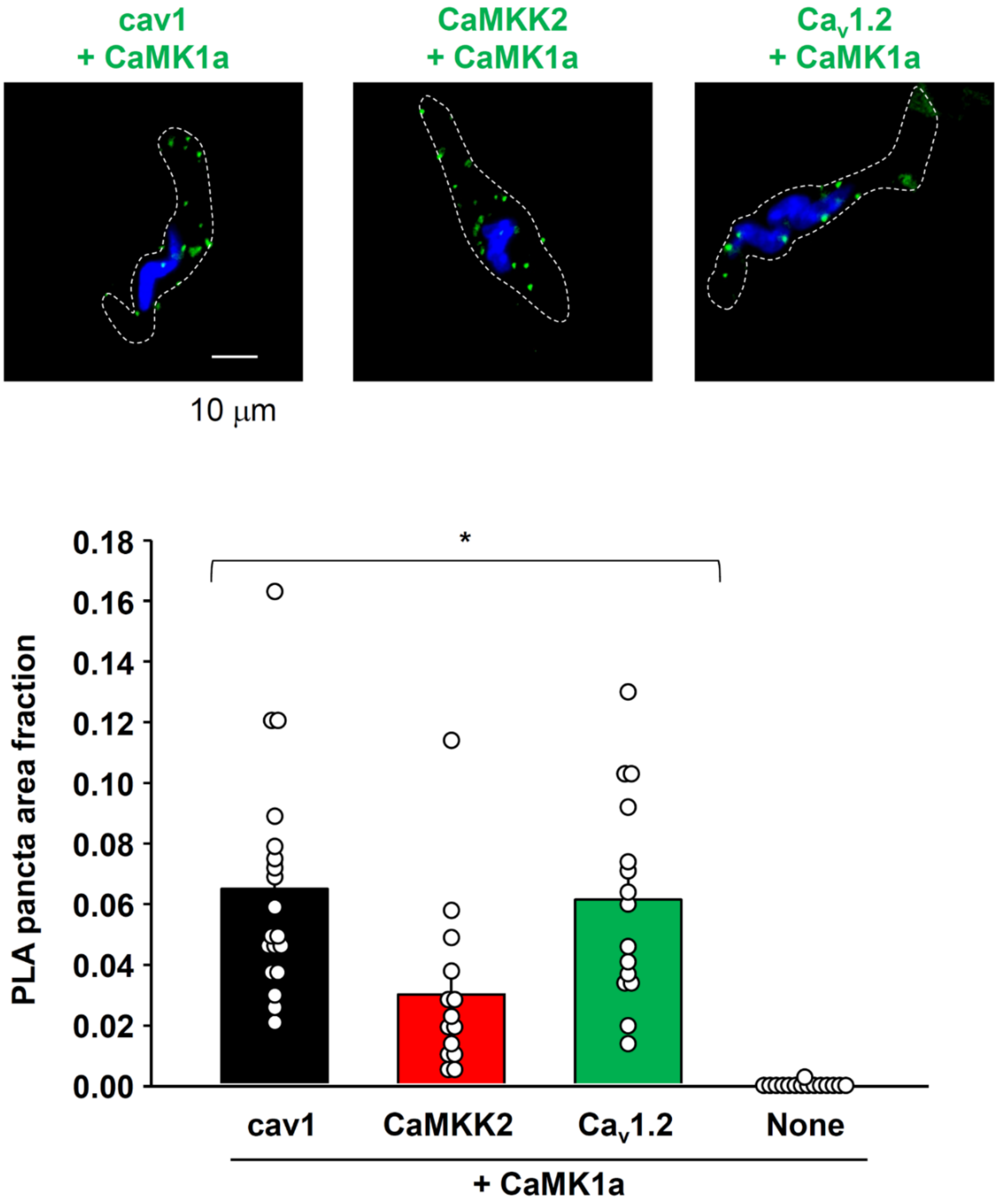

Fig. S6. CaMK1a is coupled with cav1, CaMKK2 and Ca$_V$1.2 in mesenteric artery myocytes.
PLA was performed to seek evidence for direct coupling of CaMK1a with cav1, CaMKK2 and Ca$_V$1.2 in mesenteric artery myocytes from WT mice. To obtain negative control data, myocytes were treated only with the anti-CaMK1a antibody and then subjected to PLA. Each data set was obtained from 14-19 cells. Statistical analysis was performed using one-way ANOVA followed by Dunnett test (*$p<0.05$ vs. CaMK1a).

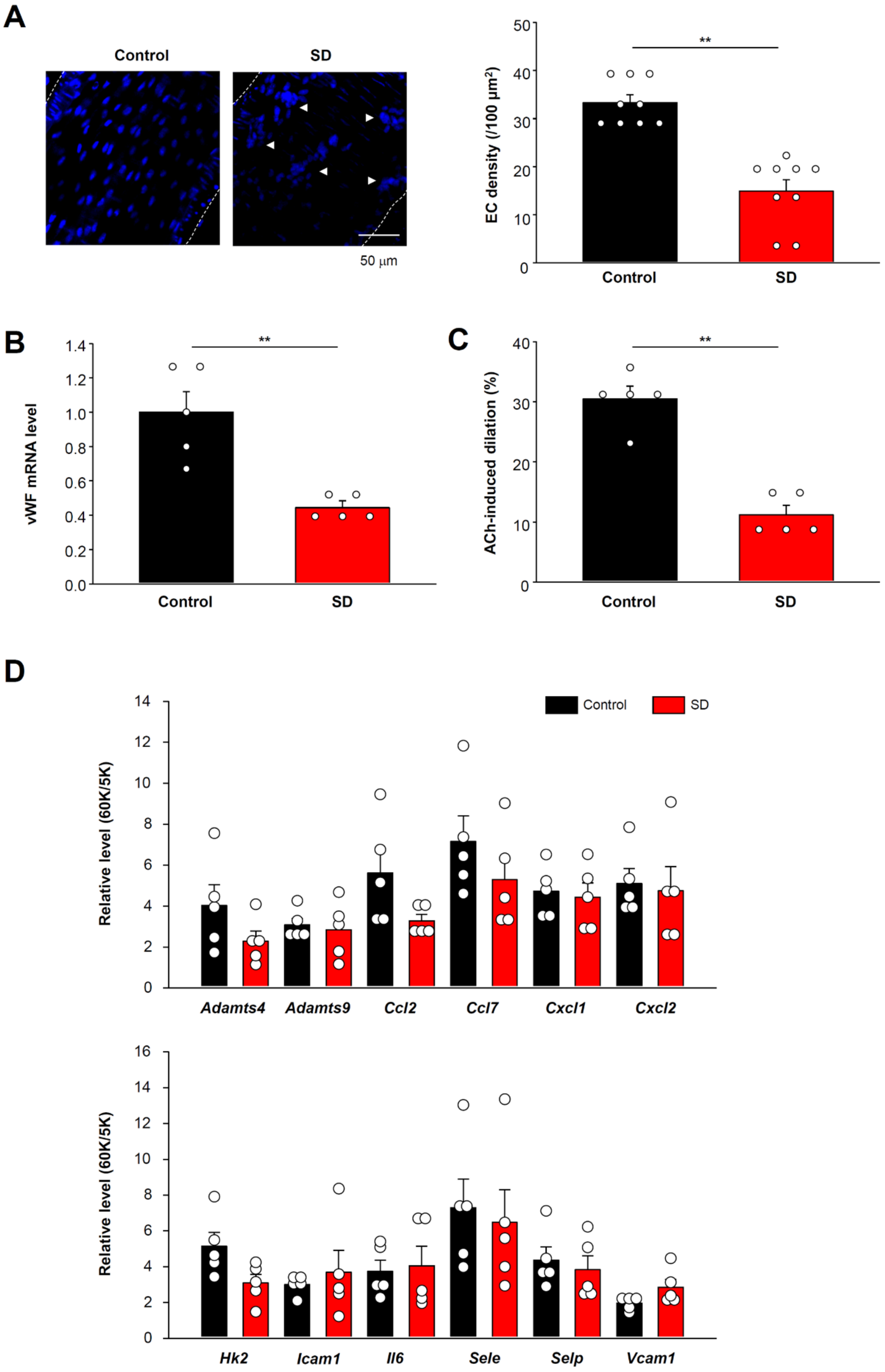

A
Control
SD
50 µm
EC density (/100 µm²)
50
40
30
20
10
0
**
Control
SD
B
vWF mRNA level
1.4
1.2
1.0
0.8
0.6
0.4
0.2
0.0
**
Control
SD
C
ACh-induced dilation (%)
40
30
20
10
0
**
Control
SD
D
Control
SD
Relative level (60K/5K)
14
12
10
8
6
4
2
0
Adamts4
Adamts9
Ccl2
Ccl7
Cxcl1
Cxcl2
Relative level (60K/5K)
16
14
12
10
8
6
4
2
0
Hk2
Icam1
Il6
Sele
Selp
Vcam1

Fig. S7. Endothelium-denuded mesenteric arteries can exhibit depolarization-induced gene transcription very similar to the pattern obtained in intact arteries.

Endothelium of mesenteric artery beds were removed by perfusing 0.2% sodium deoxycholate (SD) dissolved in ice cold PBS for 120 sec. **(A)** Confocal images of endothelial layers were obtained from control (treated with PBS) and SD groups (9 arteries from 3 mice per group). Vessels were stained with Hoechst (left). The density of endothelial cells (ECs) is compared (right). Note that the number of ECs was reduced, and ECs exhibited morphologic abnormality i.e. round, not elliptic, shape (indicated by arrowheads), and these ECs often aggregated in the SD group. For each artery, three image sections (215 μm×215 μm) were acquired and the calculated EC density values were averaged. **(B)** The gene expression of an endothelium marker, Von Willebrand Factor (VWF) was compared by qPCR. Data were obtained from 5 mice per group. **(C)** Acetylcholine (ACh)-induced vasodilation was recorded in before (Control) and after SD treatment. Vasocontraction was induced by 30 mM K^+ Hanks solution and then arterial beds were dilated by 1 μM ACh. ACh-induced vasodilation (%) was estimated from ACh-induced reversal of KCl-mediated vasocontraction. Data were obtained from 5 mice. **(D)** Gene transcription after depolarizing stimulus for 1 hour was confirmed by qPCR. Data were obtained 5 mice per group. Statistical analysis was performed using two-tailed t test (**p<0.01).

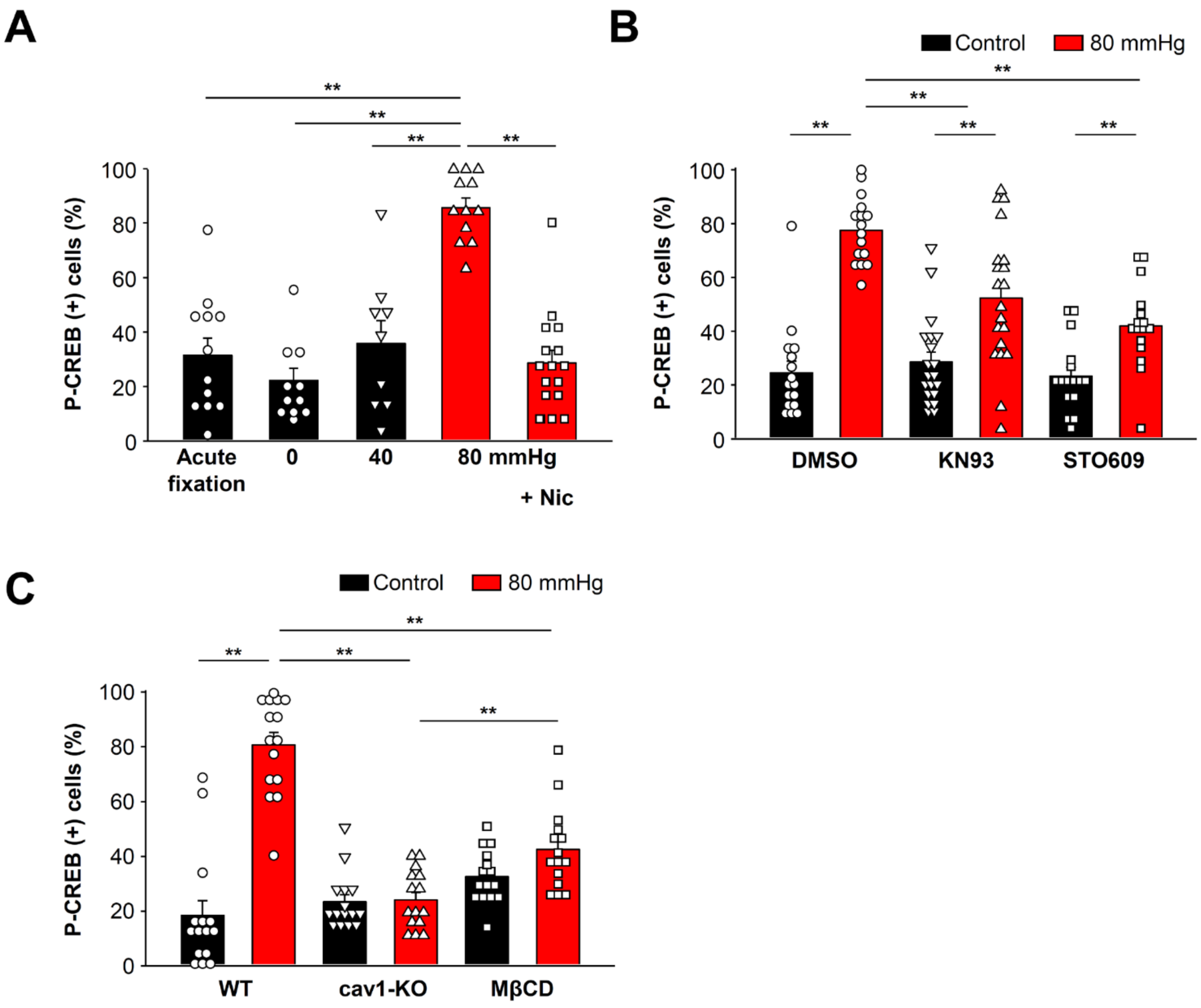

Fig. S8. Sustained increase in transmural pressure in mesenteric artery causes CREB phosphorylation in vascular myocytes by activating the $Ca_V1.2$/CaMKK2/CaMK1a axis.

The possibility that increased perfusion pressure can cause CREB phosphorylation was addressed using ex vivo model. **(A)** Mesenteric artery preparations were perfused with 5 mM K^+ solution at 0, 40 and 80 mmHg for 30 min, and then fixed and stained with P-CREB antibody. The effect of the L-type Ca^{2+} channel blocker nicardipine (Nic) on 80 mmHg-induced CREB phosphorylation also examined. The P-CREB positive myocyte ratio in vivo was estimated using acute fixation by perfusing ice cold 4% PFA from heart under deep anesthesia. Data were obtained from 9-16 arteries. **(B)** The effect of CaMK inhibitors KN93 and STO609 on 80 mmHg-induced CREB phosphorylation was examined. Data were obtained from 16-20 arteries. **(C)** High pressure (80 mmHg) was applied to mesenteric artery of WT and cav1-KO mice. Mesenteric artery treated with methyl-β-cyclodextrin (MβCD) was also utilized. Data were obtained from 15 arteries per group. Statistical analysis was performed using one-way (A) or two-way ANOVA (B and C) followed by Tukey test (**$p<0.01$).

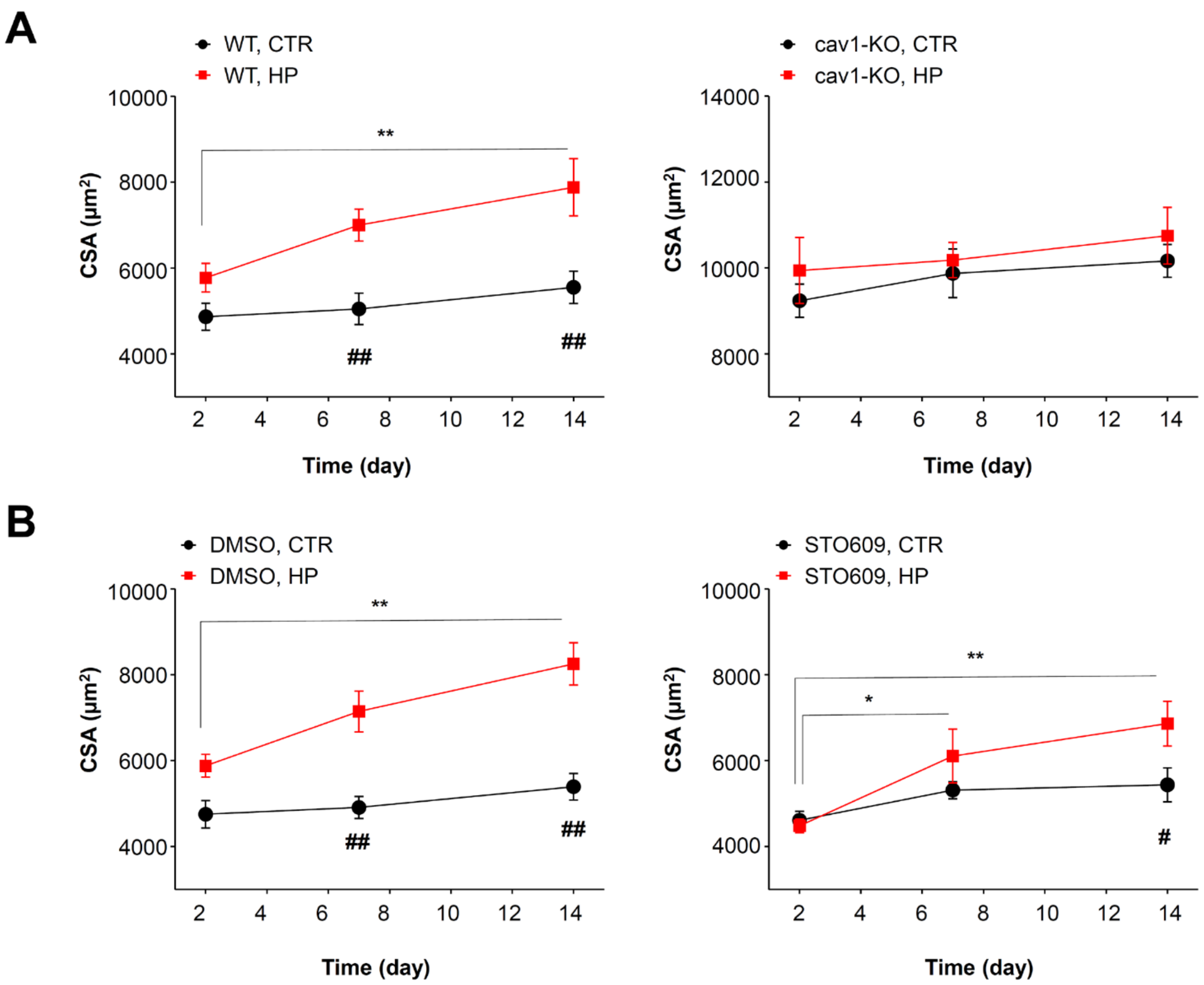

Fig. S9. Changes in cross-section area (CSA) of mesenteric arteries preloaded with high pressure.
(A) Changes in CSA of second-order mesenteric arteries from WT (left) and cav1-KO (right) mice 2, 7 and 14 days after ligation. Control vessels were second-order mesenteric arteries located along the intestine, remote from the ligated vessels. Data were collected from 4-7 arteries. **(B)** Changes in CSA of second-order mesenteric arteries from mice treated with DMSO (left) or STO609 (right) 2, 7 and 14 days after ligation was plotted. Data were collected from 5-6 arteries. Statistical analysis was performed using two-way ANOVA followed by Tukey test (* $p<0.05$, ** $p<0.01$; # $p<0.05$, ## $p<0.01$ vs. HP group at the same day).

Table S1. The list of differentially expressed genes.

Gene symbol	Gene name	Gene ID	log2.Fold_change.	qvalue
Ackr3	atypical chemokine receptor 3 [Source:MGI Symbol;Acc:MGI:109562]	ENSMUSG00000044337	1.0597	1.36E-05
Adamts4	a disintegrin-like and metallopeptidase (reprolysin type) with thrombospondin type 1 motif, 4 [Source:MGI Symbol;Acc:MGI:1339949]	ENSMUSG00000006403	2.708	4.25E-84
Adamts9	a disintegrin-like and metallopeptidase (reprolysin type) with thrombospondin type 1 motif, 9 [Source:MGI Symbol;Acc:MGI:1916320]	ENSMUSG00000030022	2.0148	4.25E-12
Adgrg1	adhesion G protein-coupled receptor G1 [Source:MGI Symbol;Acc:MGI:1340051]	ENSMUSG00000031785	1.3945	0.0021643
Agap3	ArfGAP with GTPase domain, ankyrin repeat and PH domain 3 [Source:MGI Symbol;Acc:MGI:2183446]	ENSMUSG00000023353	1.1596	2.72E-05
Akap12	A kinase (PRKA) anchor protein (gravin) 12 [Source:MGI Symbol;Acc:MGI:1932576]	ENSMUSG00000038587	1.2534	9.16E-27
Alpl	alkaline phosphatase, liver/bone/kidney [Source:MGI Symbol;Acc:MGI:87983]	ENSMUSG00000028766	1.1803	0.00079298
Arc	activity regulated cytoskeletal-associated protein [Source:MGI Symbol;Acc:MGI:88067]	ENSMUSG00000022602	1.68	2.20E-06
Arih1	ariadne RBR E3 ubiquitin protein ligase 1 [Source:MGI Symbol;Acc:MGI:1344363]	ENSMUSG00000025234	1.0438	1.85E-05
Atf4	activating transcription factor 4 [Source:MGI Symbol;Acc:MGI:88096]	ENSMUSG00000042406	1.2373	3.56E-20
Atxn7	ataxin 7 [Source:MGI Symbol;Acc:MGI:2179277]	ENSMUSG00000021738	1.23	0.0037518
Avpr1a	arginine vasopressin receptor 1A [Source:MGI Symbol;Acc:MGI:1859216]	ENSMUSG00000020123	1.1303	4.55E-07
B4galt5	UDP-Gal:betaGlcNAc beta 1,4-galactosyltransferase, polypeptide 5 [Source:MGI Symbol;Acc:MGI:1927169]	ENSMUSG00000017929	1.9999	1.04E-08
Bag3	BCL2-associated athanogene 3 [Source:MGI Symbol;Acc:MGI:1352493]	ENSMUSG00000030847	1.1922	3.72E-13
Bcl3	B cell leukemia/lymphoma 3 [Source:MGI Symbol;Acc:MGI:88140]	ENSMUSG00000053175	2.6521	2.73E-10
Bcr	breakpoint cluster region [Source:MGI Symbol;Acc:MGI:88141]	ENSMUSG00000009681	1.3652	3.96E-40
Bpnt1	bisphosphate 3'-nucleotidase 1 [Source:MGI Symbol;Acc:MGI:1338800]	ENSMUSG00000026617	1.1158	0.00065801
Btg1	B cell translocation gene 1, anti-proliferative [Source:MGI Symbol;Acc:MGI:88215]	ENSMUSG00000036478	1.0345	3.08E-09
Cbarp	calcium channel, voltage-dependent, beta subunit associated regulatory protein [Source:MGI Symbol;Acc:MGI:1354170]	ENSMUSG00000035640	1.2007	7.13E-05
Ccl2	chemokine (C-C motif) ligand 2 [Source:MGI Symbol;Acc:MGI:98259]	ENSMUSG00000035385	2.9814	0.00021177
Ccl7	chemokine (C-C motif) ligand 7 [Source:MGI Symbol;Acc:MGI:99512]	ENSMUSG00000035373	2.6699	7.30E-06
Cdc42ep4	CDC42 effector protein (Rho GTPase binding) 4 [Source:MGI Symbol;Acc:MGI:1929760]	ENSMUSG00000041598	1.73	5.12E-17
Cdkn1a	cyclin-dependent kinase inhibitor 1A (P21) [Source:MGI Symbol;Acc:MGI:104556]	ENSMUSG00000023067	1.3586	2.72E-31
Crispld2	cysteine-rich secretory protein LCCL domain containing 2 [Source:MGI Symbol;Acc:MGI:1926142]	ENSMUSG00000031825	1.4015	3.05E-223
Csf1	colony stimulating factor 1 (macrophage) [Source:MGI Symbol;Acc:MGI:1339753]	ENSMUSG00000014599	1.0836	2.43E-05
Cxcl1	chemokine (C-X-C motif) ligand 1 [Source:MGI Symbol;Acc:MGI:108068]	ENSMUSG00000029380	3.1138	3.92E-59
Cxcl16	chemokine (C-X-C motif) ligand 16 [Source:MGI Symbol;Acc:MGI:1932682]	ENSMUSG00000018920	1.873	0.0032762
Cxcl2	chemokine (C-X-C motif) ligand 2 [Source:MGI Symbol;Acc:MGI:1340094]	ENSMUSG00000058427	2.9805	4.20E-15

Dbp	D site albumin promoter binding protein [Source:MGI Symbol;Acc:MGI:94866]	ENSMUSG00000059824	-1.0969	9.50E-10
Dnajb1	DnaJ heat shock protein family (Hsp40) member B1 [Source:MGI Symbol;Acc:MGI:1931874]	ENSMUSG00000005483	2.0748	5.06E-22
Dusp14	dual specificity phosphatase 14 [Source:MGI Symbol;Acc:MGI:1927168]	ENSMUSG00000018648	1.5717	0.0016669
Dusp5	dual specificity phosphatase 5 [Source:MGI Symbol;Acc:MGI:2685183]	ENSMUSG00000034765	2.5077	0.00015392
Edn1	endothelin 1 [Source:MGI Symbol;Acc:MGI:95283]	ENSMUSG00000021367	1.1433	0.0024699
Efhd2	EF hand domain containing 2 [Source:MGI Symbol;Acc:MGI:106504]	ENSMUSG00000040659	1.4353	1.15E-37
Ell2	elongation factor RNA polymerase II 2 [Source:MGI Symbol;Acc:MGI:2183438]	ENSMUSG00000001542	1.1949	0.0014532
Emp1	epithelial membrane protein 1 [Source:MGI Symbol;Acc:MGI:107941]	ENSMUSG00000030208	1.2917	1.85E-31
Eprs	glutamyl-prolyl-tRNA synthetase [Source:MGI Symbol;Acc:MGI:97838]	ENSMUSG00000026615	1.9194	2.17E-64
Ets1	E26 avian leukemia oncogene 1, 5' domain [Source:MGI Symbol;Acc:MGI:95455]	ENSMUSG00000032035	1.7823	4.21E-07
Foxs1	forkhead box S1 [Source:MGI Symbol;Acc:MGI:95546]	ENSMUSG00000074676	1.6844	0.00032904
Gbp5	guanylate binding protein 5 [Source:MGI Symbol;Acc:MGI:2429943]	ENSMUSG00000105504	1.6845	0.0002694
Gem	GTP binding protein (gene overexpressed in skeletal muscle) [Source:MGI Symbol;Acc:MGI:99844]	ENSMUSG00000028214	1.7555	1.59E-34
Gm13889	predicted gene 13889 [Source:MGI Symbol;Acc:MGI:3652053]	ENSMUSG00000087006	1.9546	6.22E-115
Gm48878	predicted gene, 48878 [Source:MGI Symbol;Acc:MGI:6098630]	ENSMUSG00000112794	2.3573	0.0019815
Gm9973	predicted gene 9973 [Source:MGI Symbol;Acc:MGI:3642527]	ENSMUSG00000113175	1.6745	1.65E-07
Gpr4	G protein-coupled receptor 4 [Source:MGI Symbol;Acc:MGI:2441992]	ENSMUSG00000044317	2.2243	0.00059977
H3f3b	H3 histone, family 3B [Source:MGI Symbol;Acc:MGI:1101768]	ENSMUSG00000016559	1.3075	3.00E-56
Has1	hyaluronan synthase 1 [Source:MGI Symbol;Acc:MGI:106590]	ENSMUSG00000003665	1.5313	5.29E-05
Hbegf	heparin-binding EGF-like growth factor [Source:MGI Symbol;Acc:MGI:96070]	ENSMUSG00000024486	1.0841	1.57E-10
Hist1h1c	histone cluster 1, H1c [Source:MGI Symbol;Acc:MGI:1931526]	ENSMUSG00000036181	1.0529	7.56E-07
Hk2	hexokinase 2 [Source:MGI Symbol;Acc:MGI:1315197]	ENSMUSG00000000628	3.2305	1.25E-25
Hmgb2	high mobility group box 2 [Source:MGI Symbol;Acc:MGI:96157]	ENSMUSG00000054717	1.1939	0.00097572
Hr	lysine demethylase and nuclear receptor corepressor [Source:MGI Symbol;Acc:MGI:96223]	ENSMUSG00000022096	1.0624	4.84E-08
Hspa1a	heat shock protein 1A [Source:MGI Symbol;Acc:MGI:96244]	ENSMUSG00000091971	2.9825	6.48E-126
Hspa1b	heat shock protein 1B [Source:MGI Symbol;Acc:MGI:99517]	ENSMUSG00000090877	3.573	2.26E-234
Hspa5	heat shock protein 5 [Source:MGI Symbol;Acc:MGI:95835]	ENSMUSG00000026864	1.8551	6.22E-115
Hspb1	heat shock protein 1 [Source:MGI Symbol;Acc:MGI:96240]	ENSMUSG00000004951	1.3568	7.87E-104
Hspe1	heat shock protein 1 (chaperonin 10) [Source:MGI Symbol;Acc:MGI:104680]	ENSMUSG00000073676	1.4587	4.27E-05
Hsph1	heat shock 105kDa/110kDa protein 1 [Source:MGI Symbol;Acc:MGI:105053]	ENSMUSG00000029657	1.2356	2.33E-07
Icam1	intercellular adhesion molecule 1 [Source:MGI Symbol;Acc:MGI:96392]	ENSMUSG00000037405	2.9417	1.76E-91
Icosl	icos ligand [Source:MGI Symbol;Acc:MGI:1354701]	ENSMUSG00000000732	1.4928	0.00025112
Id1	inhibitor of DNA binding 1, HLH protein [Source:MGI Symbol;Acc:MGI:96396]	ENSMUSG00000042745	1.0985	5.94E-13

Id3	inhibitor of DNA binding 3 [Source:MGI Symbol;Acc:MGI:96398]	ENSMUSG00000007872	1.4255	1.69E-46
Ier3	immediate early response 3 [Source:MGI Symbol;Acc:MGI:104814]	ENSMUSG00000003541	1.2464	2.66E-18
Ier5	immediate early response 5 [Source:MGI Symbol;Acc:MGI:1337072]	ENSMUSG00000056708	1.3744	2.39E-13
Ier5l	immediate early response 5-like [Source:MGI Symbol;Acc:MGI:1919750]	ENSMUSG00000089762	1.4474	0.0016154
Il4ra	interleukin 4 receptor, alpha [Source:MGI Symbol;Acc:MGI:105367]	ENSMUSG00000030748	1.8812	2.36E-08
Il6	interleukin 6 [Source:MGI Symbol;Acc:MGI:96559]	ENSMUSG00000025746	2.422	1.96E-32
Irf1	interferon regulatory factor 1 [Source:MGI Symbol;Acc:MGI:96590]	ENSMUSG00000018899	1.0562	0.0001643
Itpk1	inositol 1,3,4-triphosphate 5/6 kinase [Source:MGI Symbol;Acc:MGI:2446159]	ENSMUSG00000057963	1.2224	3.53E-12
Jmy	junction-mediating and regulatory protein [Source:MGI Symbol;Acc:MGI:1913096]	ENSMUSG00000021690	1.0658	0.00058379
Jund	jun D proto-oncogene [Source:MGI Symbol;Acc:MGI:96648]	ENSMUSG00000071076	1.0731	4.42E-06
Lif	leukemia inhibitory factor [Source:MGI Symbol;Acc:MGI:96787]	ENSMUSG00000034394	2.064	0.0007255
Litaf	LPS-induced TN factor [Source:MGI Symbol;Acc:MGI:1929512]	ENSMUSG00000022500	1.6033	1.45E-13
Lpin1	lipin 1 [Source:MGI Symbol;Acc:MGI:1891340]	ENSMUSG00000020593	1.1171	1.45E-06
Map2k3	mitogen-activated protein kinase kinase 3 [Source:MGI Symbol;Acc:MGI:1346868]	ENSMUSG00000018932	1.1502	1.96E-05
Mapkapk2	MAP kinase-activated protein kinase 2 [Source:MGI Symbol;Acc:MGI:109298]	ENSMUSG00000016528	1.0647	1.78E-07
Mcl1	myeloid cell leukemia sequence 1 [Source:MGI Symbol;Acc:MGI:101769]	ENSMUSG00000038612	1.1695	3.40E-39
Midn	midnolin [Source:MGI Symbol;Acc:MGI:1890222]	ENSMUSG00000035621	1.0411	3.51E-11
Mt1	metallothionein 1 [Source:MGI Symbol;Acc:MGI:97171]	ENSMUSG00000031765	1.234	1.98E-50
mt-Rnr1	mitochondrially encoded 12S rRNA [Source:MGI Symbol;Acc:MGI:102493]	ENSMUSG00000064337	1.0849	5.83E-08
mt-Rnr2	mitochondrially encoded 16S rRNA [Source:MGI Symbol;Acc:MGI:102492]	ENSMUSG00000064339	1.3532	1.41E-33
Myc	myelocytomatosis oncogene [Source:MGI Symbol;Acc:MGI:97250]	ENSMUSG00000022346	1.681	4.42E-06
Ncor2	nuclear receptor co-repressor 2 [Source:MGI Symbol;Acc:MGI:1337080]	ENSMUSG00000029478	1.1405	2.75E-09
Nfil3	nuclear factor, interleukin 3, regulated [Source:MGI Symbol;Acc:MGI:109495]	ENSMUSG00000056749	1.5059	7.62E-08
Nfkbia	nuclear factor of kappa light polypeptide gene enhancer in B cells inhibitor, alpha [Source:MGI Symbol;Acc:MGI:104741]	ENSMUSG00000021025	1.6169	1.86E-36
Ngf	nerve growth factor [Source:MGI Symbol;Acc:MGI:97321]	ENSMUSG00000027859	1.3443	0.0012134
Nos3	nitric oxide synthase 3, endothelial cell [Source:MGI Symbol;Acc:MGI:97362]	ENSMUSG00000028978	1.1007	3.86E-05
Nr1d1	nuclear receptor subfamily 1, group D, member 1 [Source:MGI Symbol;Acc:MGI:2444210]	ENSMUSG00000020889	-1.8606	6.56E-24
Nup98	nucleoporin 98 [Source:MGI Symbol;Acc:MGI:109404]	ENSMUSG00000063550	1.1863	0.0010609
Odc1	ornithine decarboxylase, structural 1 [Source:MGI Symbol;Acc:MGI:97402]	ENSMUSG00000011179	1.1525	2.58E-09
Pde4b	phosphodiesterase 4B, cAMP specific [Source:MGI Symbol;Acc:MGI:99557]	ENSMUSG00000028525	1.7624	9.28E-47
Peg3	paternally expressed 3 [Source:MGI Symbol;Acc:MGI:104748]	ENSMUSG00000002265	-1.0855	0.0034048
Pim1	proviral integration site 1 [Source:MGI Symbol;Acc:MGI:97584]	ENSMUSG00000024014	1.9542	2.81E-23
Plat	plasminogen activator, tissue [Source:MGI Symbol;Acc:MGI:97610]	ENSMUSG00000031538	1.3295	8.55E-15

Plau	plasminogen activator, urokinase [Source:MGI Symbol;Acc:MGI:97611]	ENSMUSG00000021822	1.3454	1.71E-07
Plaur	plasminogen activator, urokinase receptor [Source:MGI Symbol;Acc:MGI:97612]	ENSMUSG00000046223	1.8886	4.39E-06
Por	P450 (cytochrome) oxidoreductase [Source:MGI Symbol;Acc:MGI:97744]	ENSMUSG00000005514	1.1298	8.96E-06
Pqlc1	PQ loop repeat containing 1 [Source:MGI Symbol;Acc:MGI:1914193]	ENSMUSG00000034006	1.7352	8.73E-10
Procr	protein C receptor, endothelial [Source:MGI Symbol;Acc:MGI:104596]	ENSMUSG00000027611	1.9788	2.33E-22
Ptgs2	prostaglandin-endoperoxide synthase 2 [Source:MGI Symbol;Acc:MGI:97798]	ENSMUSG00000032487	1.4719	1.92E-17
Ptpn1	protein tyrosine phosphatase, non-receptor type 1 [Source:MGI Symbol;Acc:MGI:97805]	ENSMUSG00000027540	1.2378	5.29E-05
Ptpn2	protein tyrosine phosphatase, non-receptor type 2 [Source:MGI Symbol;Acc:MGI:97806]	ENSMUSG00000024539	1.5295	0.0004601
Pvr	poliovirus receptor [Source:MGI Symbol;Acc:MGI:107741]	ENSMUSG00000040511	1.731	2.97E-07
Rcan1	regulator of calcineurin 1 [Source:MGI Symbol;Acc:MGI:1890564]	ENSMUSG00000022951	1.3662	2.45E-13
Rcc2	regulator of chromosome condensation 2 [Source:MGI Symbol;Acc:MGI:1919784]	ENSMUSG00000040945	1.307	1.26E-06
Rnd1	Rho family GTPase 1 [Source:MGI Symbol;Acc:MGI:2444878]	ENSMUSG00000054855	2.4898	2.28E-41
Rnf152	ring finger protein 152 [Source:MGI Symbol;Acc:MGI:2443787]	ENSMUSG00000047496	-1.4452	2.38E-05
Rnf19b	ring finger protein 19B [Source:MGI Symbol;Acc:MGI:1922484]	ENSMUSG00000028793	1.4218	0.0006166
Rnf217	ring finger protein 217 [Source:MGI Symbol;Acc:MGI:3610311]	ENSMUSG00000063760	1.5687	0.0010808
Rrad	Ras-related associated with diabetes [Source:MGI Symbol;Acc:MGI:1930943]	ENSMUSG00000031880	1.1934	4.44E-20
Sbno2	strawberry notch 2 [Source:MGI Symbol;Acc:MGI:2448490]	ENSMUSG00000035673	1.6347	4.14E-40
Sdc4	syndecan 4 [Source:MGI Symbol;Acc:MGI:1349164]	ENSMUSG00000017009	1.0984	7.97E-32
Sele	selectin, endothelial cell [Source:MGI Symbol;Acc:MGI:98278]	ENSMUSG00000026582	4.2624	2.36E-23
Selp	selectin, platelet [Source:MGI Symbol;Acc:MGI:98280]	ENSMUSG00000026580	2.7379	6.56E-24
Sgms2	sphingomyelin synthase 2 [Source:MGI Symbol;Acc:MGI:1921692]	ENSMUSG00000050931	2.2309	2.55E-07
Sh2b3	SH2B adaptor protein 3 [Source:MGI Symbol;Acc:MGI:893598]	ENSMUSG00000042594	1.3165	3.40E-06
Slc16a3	solute carrier family 16 (monocarboxylic acid transporters), member 3 [Source:MGI Symbol;Acc:MGI:1933438]	ENSMUSG00000025161	1.4582	3.20E-07
Slc25a25	solute carrier family 25 (mitochondrial carrier, phosphate carrier), member 25 [Source:MGI Symbol;Acc:MGI:1915913]	ENSMUSG00000026819	1.0335	8.73E-10
Slc2a1	solute carrier family 2 (facilitated glucose transporter), member 1 [Source:MGI Symbol;Acc:MGI:95755]	ENSMUSG00000028645	1.9585	1.27E-07
Slc35e4	solute carrier family 35, member E4 [Source:MGI Symbol;Acc:MGI:2144150]	ENSMUSG00000048807	1.4866	0.00066791
Slc41a3	solute carrier family 41, member 3 [Source:MGI Symbol;Acc:MGI:1918949]	ENSMUSG00000030089	1.3387	0.00023525
Slc7a2	solute carrier family 7 (cationic amino acid transporter, y+ system), member 2 [Source:MGI Symbol;Acc:MGI:99828]	ENSMUSG00000031596	-1.1298	2.81E-10
Socs3	suppressor of cytokine signaling 3 [Source:MGI Symbol;Acc:MGI:1201791]	ENSMUSG00000053113	1.61	3.22E-65
Sphk1	sphingosine kinase 1 [Source:MGI Symbol;Acc:MGI:1316649]	ENSMUSG00000061878	1.3674	8.69E-07
Sqstm1	sequestosome 1 [Source:MGI Symbol;Acc:MGI:107931]	ENSMUSG00000015837	1.13	2.10E-26

Srgn	serglycin [Source:MGI Symbol;Acc:MGI:97756]	ENSMUSG00000020077	1.0165	0.0039384
Srxn1	sulfiredoxin 1 homolog (S. cerevisiae) [Source:MGI Symbol;Acc:MGI:104971]	ENSMUSG00000032802	1.3111	4.89E-05
St3gal1	ST3 beta-galactoside alpha-2,3-sialyltransferase 1 [Source:MGI Symbol;Acc:MGI:98304]	ENSMUSG00000013846	1.1235	0.00037098
Stx11	syntaxin 11 [Source:MGI Symbol;Acc:MGI:1921982]	ENSMUSG00000039232	3.4356	4.92E-07
Tmbim1	transmembrane BAX inhibitor motif containing 1 [Source:MGI Symbol;Acc:MGI:1916910]	ENSMUSG00000006301	1.029	2.23E-09
Tnfaip2	tumor necrosis factor, alpha-induced protein 2 [Source:MGI Symbol;Acc:MGI:104960]	ENSMUSG00000021281	2.1137	5.12E-44
Tnfaip3	tumor necrosis factor, alpha-induced protein 3 [Source:MGI Symbol;Acc:MGI:1196377]	ENSMUSG00000019850	2.5895	6.96E-31
Trib1	tribbles pseudokinase 1 [Source:MGI Symbol;Acc:MGI:2443397]	ENSMUSG00000032501	1.3871	2.39E-07
Trpv4	transient receptor potential cation channel, subfamily V, member 4 [Source:MGI Symbol;Acc:MGI:1926945]	ENSMUSG00000014158	1.1583	5.86E-05
Tubb6	tubulin, beta 6 class V [Source:MGI Symbol;Acc:MGI:1915201]	ENSMUSG00000001473	1.7196	5.76E-14
Tubgcp3	tubulin, gamma complex associated protein 3 [Source:MGI Symbol;Acc:MGI:2183752]	ENSMUSG00000000759	1.2478	4.42E-07
Txnrd1	thioredoxin reductase 1 [Source:MGI Symbol;Acc:MGI:1354175]	ENSMUSG00000020250	1.194	4.36E-06
Uap1	UDP-N-acetylglucosamine pyrophosphorylase 1 [Source:MGI Symbol;Acc:MGI:1334459]	ENSMUSG00000026670	2.1732	4.27E-70
Ubc	ubiquitin C [Source:MGI Symbol;Acc:MGI:98889]	ENSMUSG00000008348	1.5107	2.47E-54
Usp37	ubiquitin specific peptidase 37 [Source:MGI Symbol;Acc:MGI:2442483]	ENSMUSG00000033364	1.4754	0.00027371
Vcam1	vascular cell adhesion molecule 1 [Source:MGI Symbol;Acc:MGI:98926]	ENSMUSG00000027962	2.3003	3.93E-122
Zc3h12a	zinc finger CCCH type containing 12A [Source:MGI Symbol;Acc:MGI:2385891]	ENSMUSG00000042677	1.6734	1.76E-07
Zfp703	zinc finger protein 703 [Source:MGI Symbol;Acc:MGI:2662729]	ENSMUSG00000085795	1.4066	0.0025267

Table S2. The sequence of primers used in this study.

Gene	sense	antisense	size (bp)
Camk1	AAGCAGGCGGAAGACATTAGG	AGTTTCTGAGTCCTCTTGTCCT	104
Camk1d	TTCGCAGTGAAGTGCATCCC	TTTCTAAGCACGGCAATCTCG	80
Camk1g	ATGGGGCGTAAGGAGGAGG	ACAGCGATCTCATTCTCTAGGC	212
Camk2a	TGCACCCGATTCACAG	TGGCAGCATACTCCTGACCA	112
Camk2b	GCACGTCATTGGCGAGGAT	ACGGGTCTCTTCGGACTGG	103
camk2d	TACATCTTGCTGGTGGGATACC	GTAAGCTCCGGCCTTGATCT	81
camk2g	ACCGACGACTACCAGCTTTTC	GCAGCATATTCCTGCGTAGATG	95
camk4	GAGAACCTCGTCCCGGATTAC	ACACAATGGATGTAGCACCCC	106
camkk1	TGGCCCACTTGGAAGAAGC	GGAGAACTTTCTAGCCGAGAGG	149
camkk2	TCATGTGTCTCTAGCCAGCC	TGACCACGATGAAGGATTCCAT	157
camk2d for splice variant	CGATTTTCCGTCACCAGAAT	TGAAAGTCCATCCCTTCCAC	
camk2g for splice variant	GCCAAGAGACGGTAGAGTGC	ACGTGGACGTGAGGGTTTAG	
Adamts4	ATGGCCTCAATCCATCCCAG	AAGCAGGGTTGGAATCTTTGC	107
Adamts9	GACTTGTGGGCAAGGTAAGG	TCAGTCTCGGGGATGTAATCTG	105
Ccl2	TTAAAAACCTGGATCGGAACCAA	GCATTAGCTTCAGATTTACGGGT	121
Ccl7	GCTGCTTTCAGCATCCAAGTG	CCAGGGACACCGACTACTG	135
Cxcl1	CTGGGATTCACCTCAAGAACATC	CAGGGTCAAGGCAAGCCTC	117
Cxcl2	CCAACCACCAGGCTACAGG	GCGTCACACTCAAGCTCTG	108
Hk2	TGATCGCCTGCTTATTCACGG	AACCGCCTAGAAATCTCCAGA	112
Icam1	GTGATGCTCAGGTATCCATCCA	CACAGTTCTCAAAGCACAGCG	213
Il6	AGACAAAGCCAGAGTCCTTCAGAGA	GCCACTCCTTCTGTGACTCCAGC	146
Sele	ATGCCTCGCGCTTTCTCTC	GTAGTCCCGCTGACAGTATGC	128
Selp	CATCTGGTTCAGTGCTTTGATCT	ACCCGTGAGTTATTCCATGAGT	105
Vcam1	AGTTGGGGATTCGGTTGTTCT	CCCCTCATTCCTTACCACCC	112
Hprt	TCAGTCAACGGGGGACATAAA	GGGGCTGTACTGCTTAACCAG	142
Ppia	GAGCTGTTTGCAGACAAAGTTC	CCCTGGCACATGAATCCTGG	125
Gapdh	CATGGCCTTCCGTGTTCCT	CCTGCTTCACCACCTTCTTGA	104

SI references

1. Suzuki Y, Yamamura H, Ohya S, & Imaizumi Y (2013) Caveolin-1 facilitates the direct coupling between large conductance Ca^{2+}-activated K^+ (BK_{Ca}) and Cav1.2 Ca^{2+} channels and their clustering to regulate membrane excitability in vascular myocytes. *J Biol Chem* 288(51):36750-36761.
2. Matsuki K*, et al.* (2018) Negative regulation of cellular Ca^{2+} mobilization by ryanodine receptor type 3 in mouse mesenteric artery smooth muscle. *Am J Physiol Cell Physiol* 315(1):C1-C9.
3. Syed AU*, et al.* (2019) Adenylyl cyclase 5-generated cAMP controls cerebral vascular reactivity during diabetic hyperglycemia. *J Clin Invest* 129(8):3140-3152.
4. Nieves-Cintron M, Amberg GC, Navedo MF, Molkentin JD, & Santana LF (2008) The control of Ca^{2+} influx and NFATc3 signaling in arterial smooth muscle during hypertension. *Proc Natl Acad Sci USA* 105(40):15623-15628.
5. Bakker EN*, et al.* (2008) Blood flow-dependent arterial remodelling is facilitated by inflammation but directed by vascular tone. *Cardiovasc Res* 78(2):341-348.
6. Saeki T, Suzuki Y, Yamamura H, Takeshima H, & Imaizumi Y (2019) A junctophilin-caveolin interaction enables efficient coupling between ryanodine receptors and BK_{Ca} channels in the Ca^{2+} microdomain of vascular smooth muscle. *J Biol Chem* 294(35):13093-13105.
